# Caffeine stabilizes Cdc25 independently of Rad3 in *S chizosaccharomyces pombe* contributing to checkpoint override

**DOI:** 10.1111/mmi.12592

**Published:** 2014-04-14

**Authors:** John P Alao, Johanna J Sjölander, Juliane Baar, Nejla Özbaki-Yagan, Bianca Kakoschky, Per Sunnerhagen

**Affiliations:** 1 Department of Chemistry and Molecular Biology, Lundberg Laboratory, University of GothenburgBox 462, SE-405 30, Göteborg, Sweden

## Abstract

Cdc25 is required for Cdc2 dephosphorylation and is thus essential for cell cycle progression. Checkpoint activation requires dual inhibition of Cdc25 and Cdc2 in a Rad3-dependent manner. Caffeine is believed to override activation of the replication and DNA damage checkpoints by inhibiting Rad3-related proteins in both *S chizosaccharomyces pombe* and mammalian cells. In this study, we have investigated the impact of caffeine on Cdc25 stability, cell cycle progression and checkpoint override. Caffeine induced Cdc25 accumulation in *S . pombe* independently of Rad3. Caffeine delayed cell cycle progression under normal conditions but advanced mitosis in cells treated with replication inhibitors and DNA-damaging agents. In the absence of Cdc25, caffeine inhibited cell cycle progression even in the presence of hydroxyurea or phleomycin. Caffeine induces Cdc25 accumulation in *S . pombe* by suppressing its degradation independently of Rad3. The induction of Cdc25 accumulation was not associated with accelerated progression through mitosis, but rather with delayed progression through cytokinesis. Caffeine-induced Cdc25 accumulation appears to underlie its ability to override cell cycle checkpoints. The impact of Cdc25 accumulation on cell cycle progression is attenuated by Srk1 and Mad2. Together our findings suggest that caffeine overrides checkpoint enforcement by inducing the inappropriate nuclear localization of Cdc25.

## Introduction

The ability to rapidly delay cell cycle progression in response to environmental and genotoxic insults, is essential for the maintenance of genomic integrity and/or cell viability. Cells have thus evolved molecular signalling pathways that sense DNA damage or environmental stress and activate cell cycle checkpoints. Understanding the interplay between the cellular environment, genome maintenance and cell cycle progression is important for understanding and/or improving the prevention, progression, and treatment of many diseases (Schumacher *et al*., 2008; Hoeijmakers, 2009).

Cell cycle progression in *Schizosaccharomyces pombe* is regulated by the activity of the cyclin-dependent kinase (CDK) Cdc2 and its regulatory cyclin Cdc13 (Lu *et al*., 2012). Negative regulation of Cdc2, and thus cell cycle progression, is enforced by the Mik1 and Wee1 kinases which phosphorylate Tyr15 to inhibit its activity. Conversely, the Cdc25 phosphatase positively regulates Cdc2 activity by dephosphorylating Tyr15 and is essential for G2/M cell cycle progression in *S. pombe* (Lu *et al*., 2012). Cdc25 levels increase throughout G2 but its activity is highly regulated by a combination of translational and post-translational mechanisms. The effective inhibition of Cdc25 and Cdc2 activity is thus essential for full activation of the DNA damage and stress activated cell cycle checkpoints (Alao and Sunnerhagen, 2008).

The central activator of the DNA damage response (DDR) pathway in *S. pombe* is the ataxia telangiectasia mutated (ATM) and ataxia – and rad related (ATR) kinase homologue Rad3, a member of the phosphatidylinositol 3 kinase-like kinase (PIKK) family (Humphrey, 2000; Lovejoy and Cortez, 2009). In response to stalled replication, *S. pombe* activates the replication or S-M checkpoint. Following its activation by stalled replication forks, Rad3 phosphorylates and activates the Cds1 kinase, a functional homologue of the mammalian Chk1 kinase (Boddy *et al*., 1998; Lindsay *et al*., 1998; Brondello *et al*., 1999). Additionally, Rad3 phosphorylates the Chk1 kinase (Chk2 in mammalian cells) in response to DNA damage occurring during the G2 phase of the cell cycle to enforce the DNA damage checkpoint. Cds1 and Chk1 phosphorylate multiple serine and threonine residues on Cdc25, thereby inactivating it (Alao and Sunnerhagen, 2008). Cds1 also induces the synthesis of Mik1, which is required for the degradation of Cdc25 remaining in the nucleus (Alao and Sunnerhagen, 2008). Rad3-induced activation of Cds1 and Chk1 requires the adaptor molecules Mrc1 and Crb2 respectively. This differential requirement for adaptor molecules ensures the cell cycle phase-specific activation of Cds1 and Chk1. Mik1 and Wee1 ensure full checkpoint activation and cell cycle arrest by phosphorylating Cdc2 on Tyr15. Mutants unable to effectively activate cell cycle checkpoints in response to DNA damage are highly sensitive to genotoxins (Alao and Sunnerhagen, 2008).

The mitogen-activated protein kinase (MAPK) pathway which regulates the environmental stress response (ESR) pathway, has also been shown to influence cell cycle progression in *S. pombe* by regulating Cdc25 activity. The p38 MAPK homologue Sty1 promotes G2/M progression in *S. pombe* by stabilizing Cdc25 (Shiozaki and Russell, 1995; Kishimoto and Yamashita, 2000). Simultaneously, exposure to environmental stress also induces the Sty1-mediated expression, phosphorylation and nuclear localization of Srk1 (Smith *et al*., 2002; Asp and Sunnerhagen, 2003). Srk1 phosphorylates the same residues as do Cds1 and Chk1 on Cdc25, resulting in its nuclear export and transient cell cycle arrest (Lopez-Aviles *et al*., 2005). Srk1 is not required for DNA damage-induced cell cycle arrest but regulates mitotic onset during the normal cell cycle by inhibiting Cdc25. Sty1 thus positively regulates Cdc25 by enhancing its stability and negatively by inhibiting its activity *via* Srk1.

The nuclear exclusion of Cdc25 plays a key role in regulating its ability. During the normal cell cycle, Cdc25 localizes predominantly in the nucleus from late G2 until the onset of mitosis. Phosphorylation of the nine regulatory serine and threonine residues within the N-terminal domain of Cdc25 creates binding sites for the 14-3-3 protein Rad24. Phosphorylation of these residues by Cds1, Chk1, or Srk1 thus results in the Rad24-mediated nuclear export of Cdc25 (Lopez-Girona *et al*., 1999; Frazer and Young, 2011; 2012). The nuclear export of Cdc25 is not, however, required for the activation of the DNA damage and replication checkpoints since *S. pombe* mutants expressing constitutively nuclear Cdc25 arrest normally (Frazer and Young, 2011; 2012). In contrast, cell cycle arrest in response to environmental stress is dependent on Srk1-mediated Cdc25 phosphorylation and nuclear export (Smith *et al*., 2002; Lopez-Aviles *et al*., 2005). The stockpiling of Cdc25 following activation of the DDR or ESR has been frequently observed and is dependent on Sty1 (Kovelman and Russell, 1996; Kishimoto and Yamashita, 2000; Alao *et al*., 2010). Sty1 thus modulates Cdc25 activity both positively through stabilization and negatively through Srk1. Recent studies have demonstrated that Cdc25 levels are not rate-limiting for cell size in *S. pombe* (Frazer and Young, 2011; 2012). Constitutively nuclear mutants are less stable than wild-type (wt) Cdc25 and are degraded in a Mik1-dependent manner during DNA damage or replication stress-induced checkpoint activation (Frazer and Young, 2011; 2012). These findings suggest that nuclear export is required for the stockpiling of Cdc25 observed in response to DDR and ESR activation (Kovelman and Russell, 1996; Kishimoto and Yamashita, 2000; Lopez-Aviles *et al*., 2005; Alao *et al*., 2010). Normal turnover of Cdc25 requires the activity of the Pub1 ubiquitin ligase (Nefsky and Beach, 1996). The Clp1 (Flp1) phosphatase negatively regulates Cdc25 activity and stability at the end of mitosis. Clp1–mediated inhibition of Cdc25 activity is required for mitotic exit, activation of the septation initiation network (SIN), and progression through cytokinesis (Trautmann *et al*., 2001; Esteban *et al*., 2004; 2008,; Mikhailov *et al*., 2004). Consequently, the elevated Cdc25 activity in *clp1*Δ and *pub1*Δ mutants slows progression through cytokinesis (Esteban *et al*., 2004; 2008; Wolfe and Gould, 2004).

The production of radical oxygen species (ROS) can result in DNA damage and activation of the DDR pathway. Similarly, DNA damage is associated with the production of ROS. Co-activation of the DDR and ESR pathways is thus a common event. It remains unclear, however, how the DDR and ESR pathways are integrated in terms of Cdc25 activity and cell cycle progression (Alao and Sunnerhagen, 2008). Activation of Sty1 enforces Cdc25 activity and mitotic progression (Shiozaki and Russell, 1995; Kishimoto and Yamashita, 2000; Alao *et al*., 2010). Mutants unable to activate the DDR pathway are driven into mitosis in a Sty1-dependent manner when exposed to ultraviolet radiation which induces both ROS production and DNA damage (Degols and Russell, 1997; Alao *et al*., 2010). Conversely, strong activation of the ESR induces Sty1-dependent Srk1 activation, Cdc25 inhibition, and cell cycle arrest (Lopez-Aviles *et al*., 2005). The activation of Sty1/Srk1 signalling by osmotic stress can thus partially compensate for the absence of DNA damage cell cycle checkpoints (Alao *et al*., 2010). In the absence of DNA damage, exposure to various stresses induces the rapid but temporary accumulation of mitotic and septated cells. This effect is dependent on both Sty1 and Cdc25 suggesting that these stresses advance progression through G2. Srk1 thus suppresses the positive effects of Sty1 on cell cycle progression. The elevated septation index may also reflect delayed progression through cytokinesis as a consequence of deregulated Cdc25 activity (Trautmann *et al*., 2001; Esteban *et al*., 2004; 2008; Mishra *et al*., 2004; Wolfe and Gould, 2004).

Caffeine is a methylxanthine commonly found in beverages such as tea and coffee, making it one of the most widely consumed neuroactive stimulants globally (Bode and Dong, 2007; Butt and Sultan, 2011). Caffeine exerts pleiotropic effects on cellular physiology and has generated much interest due to its ability to override DNA damage-induced cell cycle checkpoints (Moser *et al*., 2000; Bode and Dong, 2007). Caffeine has been shown to inhibit the activity of several PIKKs including ATM, ATR and Rad3 *in vitro* (Bode and Dong, 2007). These findings lead to the proposal that caffeine inhibits cell cycle checkpoint activation mediated by Rad3 and related PIKKs *in vivo* (Bode and Dong, 2007). This view remains controversial however, as caffeine has been shown to override DDR-activated checkpoint signalling without inhibiting ATM or ATR (Cortez, 2003). Furthermore, a direct inhibition of Rad3-induced phosphorylation of Cds1 or Chk1 in *S. pombe* cells exposed to genotoxins has not been demonstrated (Moser *et al*., 2000). Exposure to caffeine activates the Sty1-regulated ESR pathway in *S. pombe*. Furthermore, the ESR pathway is required for tolerance to caffeine in *S. pombe* (Calvo *et al*., 2009). Consequently, caffeine is likely to exert both positive and negative influences on cell cycle progression in a Cdc25-dependent manner (Alao and Sunnerhagen, 2008). Co-inhibition of p38 MAPK downstream signalling has been shown to enhance the drug-mediated inhibition of DNA damage checkpoint signalling in mammalian cells (Manke *et al*., 2005; Reinhardt *et al*., 2007). It remains unclear, however, how co-activation of Sty1 signalling influences the ability of caffeine to override DNA damage-induced checkpoints and sensitivity to genotoxic agents in *S. pombe* (Moser *et al*., 2000; Alao and Sunnerhagen, 2008; Calvo *et al*., 2009). Exposure to caffeine has been shown induce the accumulation of Cdc25A in mammalian cells. Similarly, the inhibition of ATR-Chk1 (functional homologues of Rad3 and Cds1 in mammalian cells) signalling also induces the accumulation of Cdc25A (Sørensen *et al*., 2003; 2004). Cdc25A degradation is required for activation of the S-phase checkpoint in mammalian cell lines. Hence, caffeine-induced stabilization of Cdc25A, rather than inhibition of ATM/ATR signalling, may underlie its ability to override DNA damage checkpoints (Cortez, 2003; Sørensen *et al*., 2003; 2004; 2005; Mochida and Yanagida, 2006). In addition, caffeine has also been shown to induce Cdc25B accumulation in mammalian cells (Varmeh and Manfredi, 2009). The effect of exposure to caffeine or *rad3*/*cds1* deletion on Cdc25 stability in *S. pombe* has not been previously reported. Furthermore, the impact of caffeine-mediated Sty1 activation on its ability to override DNA damage checkpoint activation has not been investigated.

In this study, we have investigated the effect of caffeine on Cdc25 stability, cell cycle progression and DNA damage/replication checkpoint activation in *S. pombe*. We also investigated the impact of Sty1 co-activation on the ability of caffeine to override DNA damage/replication checkpoints in this organism. Our findings demonstrate that caffeine induces Cdc25 accumulation independently of Rad3. Caffeine-induced Cdc25 accumulation was associated with delayed progression through cytokinesis. Interestingly, the ability of caffeine to override checkpoint signalling was not associated with the inhibition of Rad3. The ability of caffeine to override checkpoint signalling was dependent on Cdc25 expression. Lastly, our findings indicate that co-activation of Sty1 attenuates the ability of caffeine to override DNA damage checkpoint signalling.

## Results

### Caffeine induces Cdc25 accumulation independently of Rad3 and Cds1

**Figure 1:**
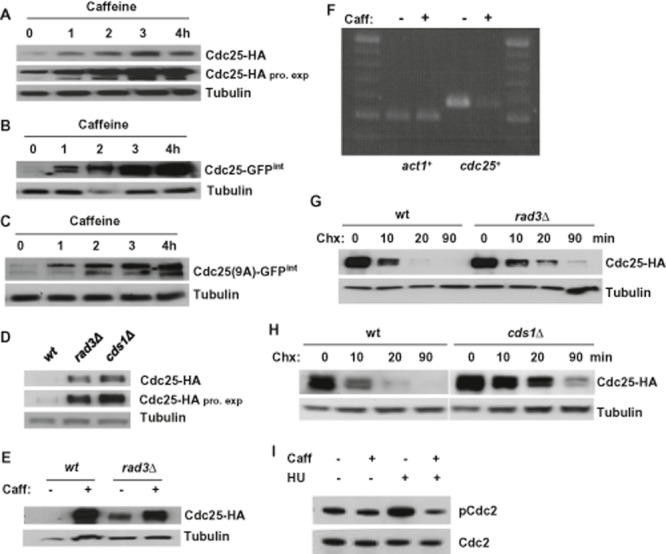
Caffeine induces Cdc25 accumulation in *S . pombe*.A. Strains expressing Cdc25-HA were incubated with 10 mM caffeine and harvested at the indicated time points. Total protein lysates were resolved by SDS-PAGE and Cdc25 detected using antibodies directed against HA. Tubulin was used to monitor gel loading. Pro exp. = prolonged exposure.B and C. Strains expressing Cdc25–GFP^int^ or Cdc25^(9A)^–GFP^int^ were treated as in A.D. Total protein lysates from log phase wt, *rad3*Δ and *cds1*Δ cultures were resolved by SDS-PAGE and probed using antibodies directed against HA. Tubulin was used to monitor gel loading. Pro exp. = prolonged exposure.E. Wt and *rad3*Δ strains were grown with or without 10 mM caffeine for 24 h and analysed as in D.F. Total RNA was extracted from wt cells exposed to 10 mM caffeine for 2 h. The expression levels of *cdc25^+^* were analysed by RT-PCR.G. Wt and *rad3*Δ cells expressing Cdc25-HA were grown to log phase, exposed to 100 μg ml^−1^ Chx and harvested at the indicated time points. Cell lysates were treated as in A.H. Wt and *cds1*Δ cells expressing Cdc25-HA were grown to log phase and treated as in G.I. Cells expressing HA-tagged Chk1 were exposed to 20 mM HU with or without 10 mM caffeine. Cells were pre-treated with HU for 2 h and then cultured with or without caffeine for a further 2 h. Total lysates were probed with antibodies directed against phosphorylated and total Cdc2.

Exposure to 10 mM caffeine resulted in rapid accumulation of HA-tagged Cdc25 under control of the endogenous promoter in log phase *S. pombe* cells (Fig. 1A). We obtained similar results by exposing cells expressing GFP-tagged Cdc25 under control of the endogenous promoter (Cdc25–GFP^int^) (Frazer and Young, 2011; 2012), or Myc-tagged Cdc25 under control of the endogenous promoter, to caffeine (Fig. 1B and Supplementary Fig. S1A). Caffeine also induced accumulation of Cdc25^(9A)^–GFP^int^ (Frazer and Young, 2011; 2012), in which the nine N-terminal serine/threonine residues phosphorylated by Cds1, Chk1 and Srk1 are mutated to alanine (Fig. 1C). Interestingly, Cdc25 levels were also constitutively elevated in *rad3*Δ and *cds1*Δ mutants (Fig. 1D). Rad3 and Cds1 thus appear to regulate Cdc25 stability in *S. pombe* as reported for the functional homologues, ATR, Chk1 and Cdc25A in mammalian cells (Sørensen *et al*., 2004). Caffeine also induced further accumulation of Cdc25 in *rad3*Δ mutants (Fig. 1E). The ability of caffeine to induce Cdc25 accumulation is therefore independent of Rad3 inhibition. The accumulation of Cdc25 observed in caffeine-treated cells and in *rad3*Δ and *cds1*Δ mutants was not due to enhanced transcription. In fact, *cdc25^+^* mRNA expression was suppressed under these conditions (Fig. 1F and Supplementary Fig. S1C). The stability of Cdc25 was increased in both *rad3*Δ and *cds1*Δ mutants (Fig. 1G and H, Supplementary Fig. S1D). Thus, caffeine or deletion of the *rad3^+^* or *cds1^+^* genes stabilizes Cdc25 by post–translational mechanisms. Caffeine suppressed Cdc2 Tyr15 phosphorylation in untreated log phase cultures, as well as in cultures exposed to hydroxyurea (HU) (Fig. 1I). These findings suggest that caffeine partly suppresses Cdc2 Tyr15 phosphorylation by stabilizing Cdc25.

### Effect of caffeine on cell cycle progression in *S . pombe*

**Figure 2:**
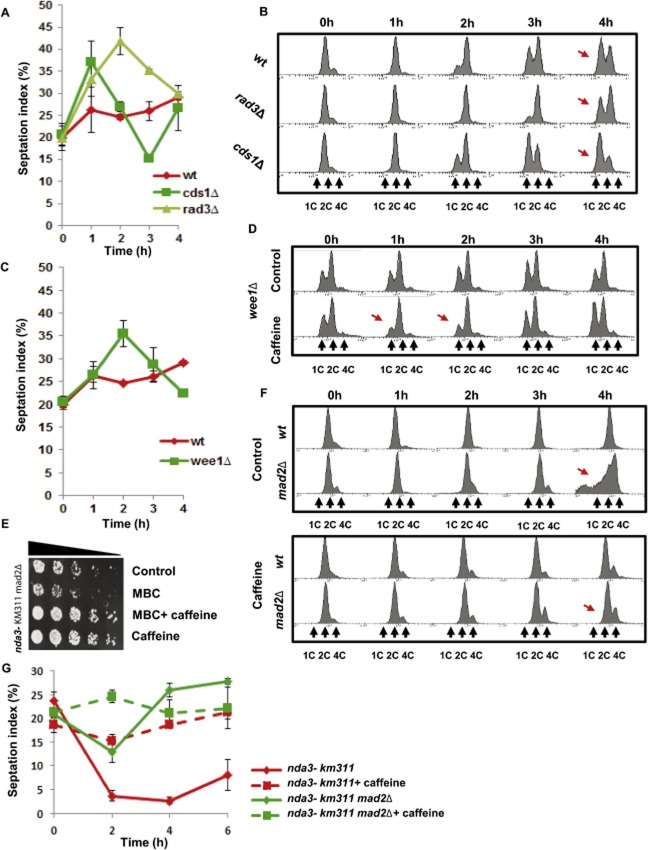
Caffeine modulates cell progression in *S . pombe*.A. Wt, *rad3*Δ, and *cds1*Δ strains were exposed to 10 mM caffeine. Samples were harvested at the indicated time points and fixed in 70% ethanol. Cells were stained with aniline blue and the septation index determined by fluorescence microscopy. At least 200 cells were counted for each time point. Error bars represent the mean of at least three independent experiments ± S.E.B. Wt, *rad3*Δ, and *cds1*Δ strains were simultaneously exposed to 20 mM HU and 10 mM caffeine. Samples were harvested at the indicated time points, fixed in 70% ethanol and analysed by FACS. Arrows indicate differential rates of cell cycle progression.C and D. *wee1*Δ mutants were treated as in A and B. Arrows indicate differential rates of cell cycle progression. Wt septation index from A was included for clarity.E. *nda3-KM311 mad2*Δ mutants were incubated at 18°C untreated (Control), treated with 50 μg ml^−1^ MBC, 50 μg ml^−1^ MBC and 10 mM caffeine, or 10 mM caffeine for 4 h. Equal cell numbers were spotted onto YES agar plates and incubated for 3 days.F. *nda3-KM311* and *nda3-KM311 mad2*Δ mutants were incubated at 18°C in the absence (top panel) or presence (bottom panel) of 10 mM caffeine. Samples were harvested at the indicated time points and analysed by FACS.G. *nda3-KM311* and *nda3-KM311 mad2*Δ were treated as in F. Samples were harvested at the indicated time points and fixed in 70% ethanol. Cells were stained with aniline blue and the septation index determined by fluorescence microscopy. At least 200 cells were counted for each time point. Error bars represent the mean of at least three independent experiments ± S.E.

The effect of caffeine-mediated Cdc25 accumulation and Cdc2 phosphorylation on the cell cycle kinetics of *S. pombe* was investigated. Exposure of wt log phase cultures to 10 mM caffeine did not significantly affect cell division kinetics (Fig. 2A). However, exposure of *cds1*Δ mutants to caffeine did induce a significant increase in the percentage of septating cells within 1 h of exposure, followed by a transient decline in the septation index between 3 and 4 h after exposure (Fig. 2A). A similar increase in the septation index was observed when *rad3*Δ mutants were exposed to caffeine. In contrast to *cds1*Δ mutants however, the caffeine-induced increase in the septation index was sustained (Fig. 2A). Caffeine induces the accumulation of Cdc25 independently of Rad3 (Fig. 1). The suppression of Cdc2 activity is required for exiting mitosis and progression through cytokinesis. Modest increases in Cdc25 activity will thus drive cells through mitosis and cytokinesis. In contrast, high levels of Cdc25 activity will advance entry into mitosis but delay progression through cytokinesis (Trautmann *et al*., 2001; Esteban *et al*., 2004; 2008; Mishra *et al*., 2004; Wolfe and Gould, 2004). To test this possibility, FACS analysis was used to monitor the progression through cytokinesis of cells exposed simultaneously to caffeine and HU. As the cells pass through cytokinesis, they accumulate as a 1C population due to HU-induced nucleotide depletion (Fig. 2B). When *rad3*Δ mutants were exposed to caffeine, their progression through cytokinesis was clearly delayed relative to the wt strain (Fig. 2A and B). Consistent with the results in Fig. 2A, *cds1*Δ mutants were advanced through both mitosis and cytokinesis (Fig. 2B). We also observed that caffeine influenced cell cycle progression to a similar degree in strains expressing Cdc25–GFP^int^ or Cdc25^(9A)^–GFP^int^ (Supplementary Fig. S3A). Further analyses demonstrated a simultaneous increase in both the number of binucleates and the septation index (Supplementary Fig. S2C). These observations suggest a general decrease in the progression from mitosis and cytokinesis in these strains following exposure to caffeine.

To further examine the effect of caffeine on cell cycle progression, we monitored its effects on the kinetics of cell division in *wee1*Δ mutants. The absence of Wee1 results in constitutively high Cdc2 activity that advances the entry of shortened cells into mitosis (Russell and Nurse, 1987). The effect of caffeine on the septation index of *wee1*Δ mutants was similar to that observed in *rad3*Δ mutants (Fig. 2A and C). The short length at division of *wee1*Δ mutants imposes a size constraint that delays progression into S phase (Nurse, 1990). Unlike wt cells in log phase, *wee1*Δ mutants spend a significantly longer amount of time in G1 and can be monitored by FACS analysis (Fig. 2D). Hence, although a G1 population is not detectable in wt cells under normal growth conditions, *wee1*Δ mutants can be used to monitor G1- to S-phase progression. Exposure to caffeine induced a rapid decline in the G1 population of *wee1*Δ mutants 1–2 h after exposure following by a gradual increase at 3–4 h (Fig. 2D). Caffeine thus induces cell cycle progression in *S. pombe wee1*Δ mutants.

To determine if caffeine delays progression through cytokinesis, its effect on cell division in *nda3-KM311^CS^* mutants was examined. The *S. pombe nda3^+^* gene encodes β-tubulin, which is unable to polymerize into microtubules at the restrictive temperature (18–20°C) in *nda3-KM311^CS^* mutants. The failure to form mitotic spindles at the restrictive temperature prevents ‘satisfaction’ of the spindle checkpoint and results in metaphase arrest (Hiraoka *et al*., 1984). Mutants lacking *mad2^+^* are unable to activate the spindle checkpoint and attempt mitosis without mitotic spindles, resulting in chromosome missegregation and loss of viability (Fig. 2 E–G; He *et al*., 1997). Caffeine (10 mM) prevented chromosome missegregation and loss of viability in *mad2*Δ mutants grown at the restrictive temperature. Caffeine similarly suppressed the sensitivity of *mad2*Δ mutants to the microtubule depolymerizing agent methylbenzimidazol-2yl carbamate (carbendazim/MBC) (Fig. 2E). As a culture of *nda3-KM311^CS^* mutants arrests in metaphase at the restrictive temperature, a corresponding decrease in the septation index can be observed. This decline in the septation index was not observed in *nda3-KM311^CS^ mad2*Δ double mutants, which continue to progress through mitosis (Fig. 2G) (Hiraoka *et al*., 1984; He *et al*., 1997). Caffeine abolished this decline in the septation index in the *nda3-KM311^CS^* mutant, suggesting it either overrides the spindle checkpoint or delays the progression of septating cells through cytokinesis (Fig. 2G). Our finding that caffeine prevents chromosome missegregation and loss of viability in *mad2*Δ mutants clearly supports the latter explanation (Fig. 2E–G). A similar delay in the progression through cytokinesis was observed in *nda3-KM311^CS^ clp1*Δ and *nda3-KM311^CS^ pub1*Δ mutants, which harbour elevated Cdc25 levels, when grown at the restrictive temperature (Supplementary Fig. S2D and E). Caffeine thus exerts both positive and negative effects on cell cycle progression in *S. pombe*.

### Mad2 delays caffeine-induced cell cycle progression in *S . pombe*

**Figure 3:**
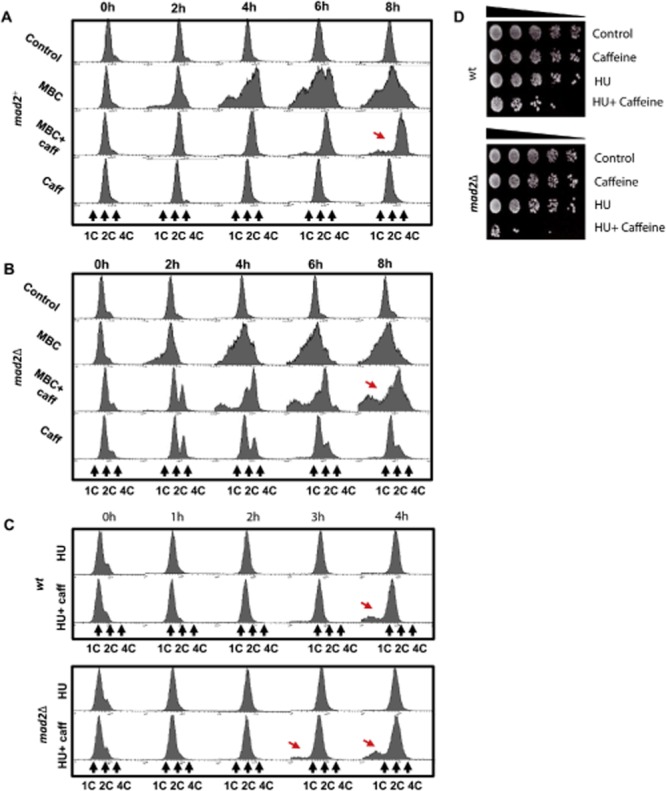
Mad2 attenuates the effect of caffeine on the cell cycle progression.A and B. *nda3-KM311* and *nda3-KM311 mad2*Δ strains were incubated at 30°C in the presence of 50 μg ml^−1^ MBC or 10 mM caffeine alone or in combination. Samples were harvested at the indicated time points and analysed by FACS. Arrows indicate differential rates of cell cycle progression.C. Wt and *mad2*Δ mutants were incubated for 2 h with 20 mM HU (0–2 h) and then incubated for a further 2 h (2–4 h) in the presence or absence of 10 mM caffeine. Samples were treated as in A. Arrows indicate differential rates of cell cycle progression.D. Wt and *mad2*Δ strains were incubated with 20 mM HU alone or in combination with 10 mM caffeine at 30 for 8 h. Equal cell numbers were spotted onto YES agar plates and incubated at 30°C for 3 days.

Exposure to caffeine activates the ESR pathway regulated through the MAPK Sty1 (Calvo *et al*., 2009). We previously demonstrated that exposure to osmotic stress, which similarly activates Sty1, partially delays progression through mitosis in a Mad2-dependent manner (Alao *et al*., 2010). The effect of Mad2 on caffeine-induced cell cycle progression was thus investigated. *S. pombe* cells partially delay progression through mitosis following exposure to the microtubule depolymerizing agent MBC (50 μg ml^−1^) in a Mad2-dependent manner (He *et al*., 1997; Castagnetti *et al*., 2010) (Fig. 3A and B). Caffeine (10 mM) suppressed MBC-induced chromosome missegregation. *mad2^+^* cells exposed to MBC and caffeine had a 4C DNA content, indicating that caffeine delays progression through cytokinesis (Fig. 3A). The rate and magnitude of MBC-induced chromosome missegregation was greater in *mad2*Δ mutants relative to *mad2^+^* cells (Fig. 3A and B). Caffeine suppressed chromosome missegregation less efficiently in *mad2*Δ mutants although DNA replication was not delayed (Fig. 3A and B). Cell cycle progression, as indicated by the increased 4C DNA content, was also advanced in *mad2*Δ mutants relative to *mad2^+^* cells exposed to caffeine alone (Fig. 3A and B).

We next compared the rate of cell cycle progression in wt and *mad2*Δ mutants arrested with 20 mM HU for 2 h and subsequently exposed to 10 mM caffeine. The rate and degree of chromosome missegregation was slightly higher in *mad2*Δ mutants relative to wt cells (Fig. 3C). Furthermore, caffeine was more effective at enhancing sensitivity to HU in *mad2*Δ mutants (Fig. 3D). The ability of caffeine to override the HU induced replication (S-M) checkpoint is thus enhanced by the *mad2* deletion. Together, these observations suggest that Mad2 attenuates the ability of caffeine to advance cell cycle progression. They also provide further evidence that caffeine can advance entry into mitosis but slows progression through cytokinesis. Concurrently, caffeine partly compensates for the lack of a spindle checkpoint by delaying progression through cytokinesis (Figs 2E–G and 3A–D).

### Caffeine advances cell cycle progression through Cdc25

**Figure 4:**
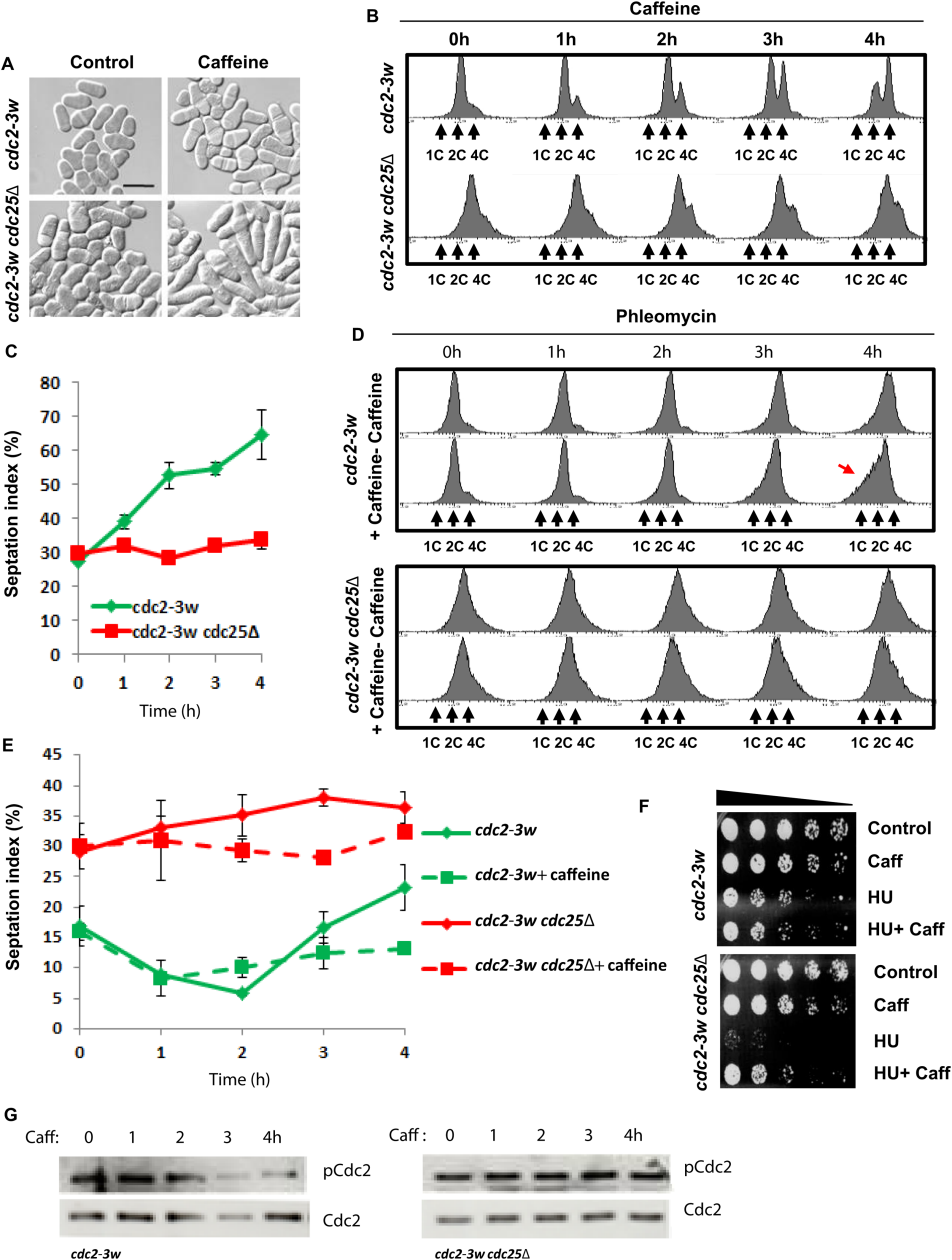
Caffeine promotes cell cycle progression in a Cdc25-dependent manner.A. *cdc2-3w* and *cdc2-3w cdc25*Δ strains were incubated with 10 mM caffeine for 4 h, fixed in 70% ethanol. Scale bar, 20 μM.B. *cdc2-3w* and *cdc2-3w cdc25*Δ strains were incubated with 10 mM caffeine. Samples were harvested at the indicated time points and analysed by FACS.C. Strains in B were stained with aniline blue and the septation index determined by fluorescence microscopy. At least 200 cells were counted for each time point. Error bars represent the mean of at least three independent experiments ± S.E.D. *cdc2-3w* and *cdc2-3w cdc25*Δ strains were incubated with 10 μg ml^−1^ phleomycin alone or in combination with 10 mM caffeine. Samples were harvested at the indicated time points and analysed by FACS. Red arrow indicates chromosome missegregation.E. *cdc2-3w* and *cdc2-3w cdc25*Δ strains were incubated with 10 μg ml^−1^ phleomycin alone or in combination with 10 mM caffeine. Samples harvested at the indicated time points were stained with aniline blue and the septation index determined by fluorescence microscopy. Error bars represent the mean of at least three independent experiments ± S.E.F. *cdc2-3w* and *cdc2-3w cdc25*Δ strains were incubated for 3 h with 20 mM HU and then incubated for a further 3 h in the presence or absence of 10 mM caffeine. Equal cell numbers were spotted onto YES agar plates and incubated at 30°C for 3 days.G. *cdc2-3w* and *cdc2-3w cdc25*Δ strains were incubated with 10 mM caffeine and harvested at the indicated time points. Total lysates were probed with antibodies directed against phosphorylated and total Cdc2.

The *cdc2-3w* allele abolishes the requirement for Cdc25-mediated entry into mitosis and activation of the replication checkpoint. Mutants carrying the *cdc2-3w* allele remain under the control of Wee1 phosphorylation and deletion of Cdc25 results in increased cell length (Enoch *et al*., 1992; Basi and Enoch, 1996) (Fig. 4A). To further investigate the role of Cdc25 in mediating the effects of caffeine, we compared its impact on cell cycle progression in *cdc2-3w* and *cdc2-3w cdc25*Δ mutants. Exposure of *cdc2-3w* mutants to 10 mM caffeine induced the sustained accumulation of septated cells with a 4C DNA content. In contrast, caffeine exerted only minor effects on cell cycle progression in *cdc2-3w cdc25*Δ mutants (Fig. 4B and C). A simultaneous increase in both the number of binucleates and the septation index was observed (Supplementary Fig. S4B). It remains unclear if the slight increase in the > 4C DNA peak reflects a delay in the cell cycle progression of the *cdc2-3w cdc25*Δ mutant. Exposure of *cdc2-3w* mutants to caffeine in the presence of latrunculin B (Lat B) (in order to inhibit cytokinesis), demonstrated that progression through mitosis and the subsequent S phase was only moderately delayed (Supplementary Fig. S4C). Caffeine thus delays progression through cytokinesis in *cdc2-3w* mutants. In contrast, caffeine did inhibit mitotic progression in *cdc2-3w cdc25*Δ mutants under similar conditions (Supplementary Fig. S4C). We next compared the ability of caffeine to enhance sensitivity to phleomycin in *cdc2-3w* and *cdc2-3w cdc25*Δ mutants. Caffeine overrode the partial checkpoint arrest in *cdc2-3w* mutants exposed to 10 μg ml^−1^ phleomycin, resulting in the accumulation of cells with missegregated chromosomes (Fig. 4D). In *cdc2-3w cdc25*Δ mutants, caffeine blocked the phleomycin-induced increase of the septation index (Fig. 4E). Interestingly, *cdc2-3w cdc25*Δ but not *cdc2-3w* mutants became elongated following exposure to caffeine (Fig. 4A and Supplementary Fig. S4A). These observations suggested that caffeine inhibits cell cycle progression in the absence of Cdc25. To test this possibility, we compared the effect of caffeine on HU sensitivity in *cdc2-3w* and *cdc2-3w cdc25*Δ mutants. Co-exposure to 10 mM caffeine did not further enhance the sensitivity of *cdc2-3w* mutants to 20 mM HU (Fig. 4F). In marked contrast, caffeine significantly suppressed the sensitivity of *cdc2-3w cdc25*Δ mutants to HU (Fig. 4F). Furthermore, exposure to 10 mM caffeine induced Cdc2 Tyr15 dephosphorylation in *cdc2-3w* but not *cdc2-3w cdc25*Δ mutants (Fig. 4G). These findings indicate that Cdc25 mediates the ability of caffeine to promote cell cycle progression. Our findings demonstrate that caffeine exerts positive and negative effects on cell cycle progression. In the absence of Cdc25, the negative effects predominate and thus slow progression through the cell cycle. Consequently, caffeine exerts opposing effects on cell cycle progression in *cdc2-3w* and *cdc2-3w cdc25*Δ mutants.

### Srk1 counteracts the ability of caffeine to override the replication checkpoint

**Figure 5:**
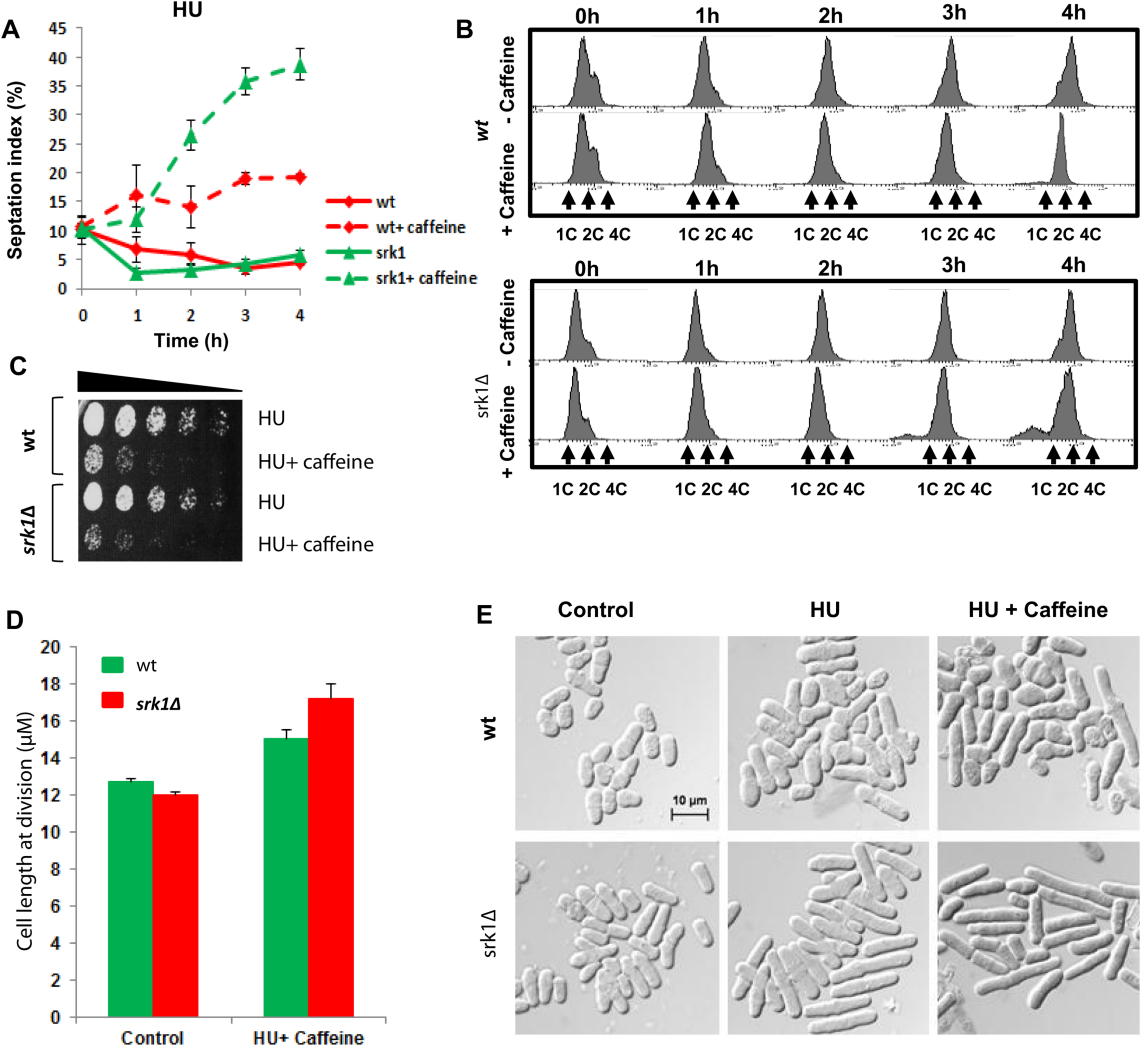
Srk1 suppresses caffeine-induced checkpoint override.A. Wt and *srk1Δ* strains were incubated with 20 mM HU for 2 h and then for a further 4 h in the presence or absence of 10 mM caffeine. Samples harvested at the indicated time points were stained with aniline blue and the septation index determined by fluorescence microscopy. Error bars represent the mean of at least three independent experiments ± S.E.B. Samples from A were analysed by FACS.C. Wt and *srk1*Δ strains were incubated with 20 mM HU for 4 h in the presence or absence of 10 mM caffeine. Equal cell numbers were spotted onto YES agar plates and incubated at 30°C for 3 days.D. Wt and *srk1*Δ strains were incubated with 20 mM HU for 2 h and then for a further 2 h in the presence of 10 mM caffeine. Cell length at division was determined by microscopy. At least 30 cells were counted for each sample.E. Wt and *srk1*Δ strains were incubated with 20 mM HU for 2 h and then for a further 2 h in the presence or absence of 10 mM caffeine. Cells were fixed in 70% ethanol and examined by microscopy. Scale bar, 10 μm.

The ability of caffeine to override cell cycle checkpoints is dependent on Cdc25. However, exposure to caffeine also activates Sty1 which delays cell cycle progression by inducing and stabilizing the Srk1 kinase (Calvo *et al*., 2009) (Supplementary Fig. S5A and B). Srk1 regulates entry into mitosis by negatively regulating Cdc25 activity during the normal cell cycle and following activation of the ESR (Smith *et al*., 2002; Lopez-Aviles *et al*., 2005; Alao *et al*., 2010). Co-activation of Srk1 may thus inhibit the ability of caffeine to override cell cycle checkpoints. Hence, we investigated the ability of Srk1 to attenuate the ability of caffeine to override the replication and DNA damage checkpoints. As previously reported, caffeine overrides the HU-induced replication checkpoint in *S. pombe* (Wang *et al*., 1999; Moser *et al*., 2000) (Fig. 5A). Wt cells and *srk1*Δ mutants were exposed to 20 mM HU for 2 h and then incubated for a further 4 h in the absence or presence of 10 mM caffeine. Under these conditions, wt cells attempt mitosis with incompletely replicated DNA resulting in chromosome missegregation and a loss of viability (Wang *et al*., 1999; Moser *et al*., 2000) (Fig. 5A–C). Remarkably, the ability of caffeine to override the replication checkpoint was greatly enhanced in *srk1*Δ mutants (Fig. 5A–C). Caffeine also induced Srk1 accumulation in the presence of HU (Supplementary Fig. S2B). The percentage of septated cells was higher in *srk1*Δ mutants relative to wt cells, suggesting an increased rate of progression through mitosis (Fig. 5A). The rate and degree of chromosome missegregation was also higher in *srk1*Δ mutants. The ability of caffeine to enhance sensitivity to HU was also elevated in *srk1*Δ mutants relative to wt cells (Fig. 5B and C). We cannot rule out, however, that elevated Cdc25 activity in the *srk1*Δ mutants also delays progression through cytokinesis and thus an increase in the septation index (Supplementary Fig. S5C).

Interestingly, the cell length at division in *srk1*Δ mutants exposed to HU and caffeine was longer than that of wt cells, as opposed to the situation in unexposed cells when *srk1Δ* mutants are slightly shorter (Fig. 5D and E). In *S. pombe*, the timing of mitosis is determined by cell length (Nurse, 1975; Russell and Nurse, 1987). Given the faster rate of cell cycle progression observed in *srk1*Δ mutants, this seemed counterintuitive. Previous studies have demonstrated that Srk1 stabilizes Cdc25 levels in *S. pombe* (Lopez-Aviles *et al*., 2005). Mutants lacking Srk1 may thus proceed initially from G2 into mitosis more slowly than wt cells when exposed to caffeine in the presence of HU as Cdc25 accumulates.

### Caffeine mediates checkpoint override by stabilizing Cdc25

**Figure 6:**
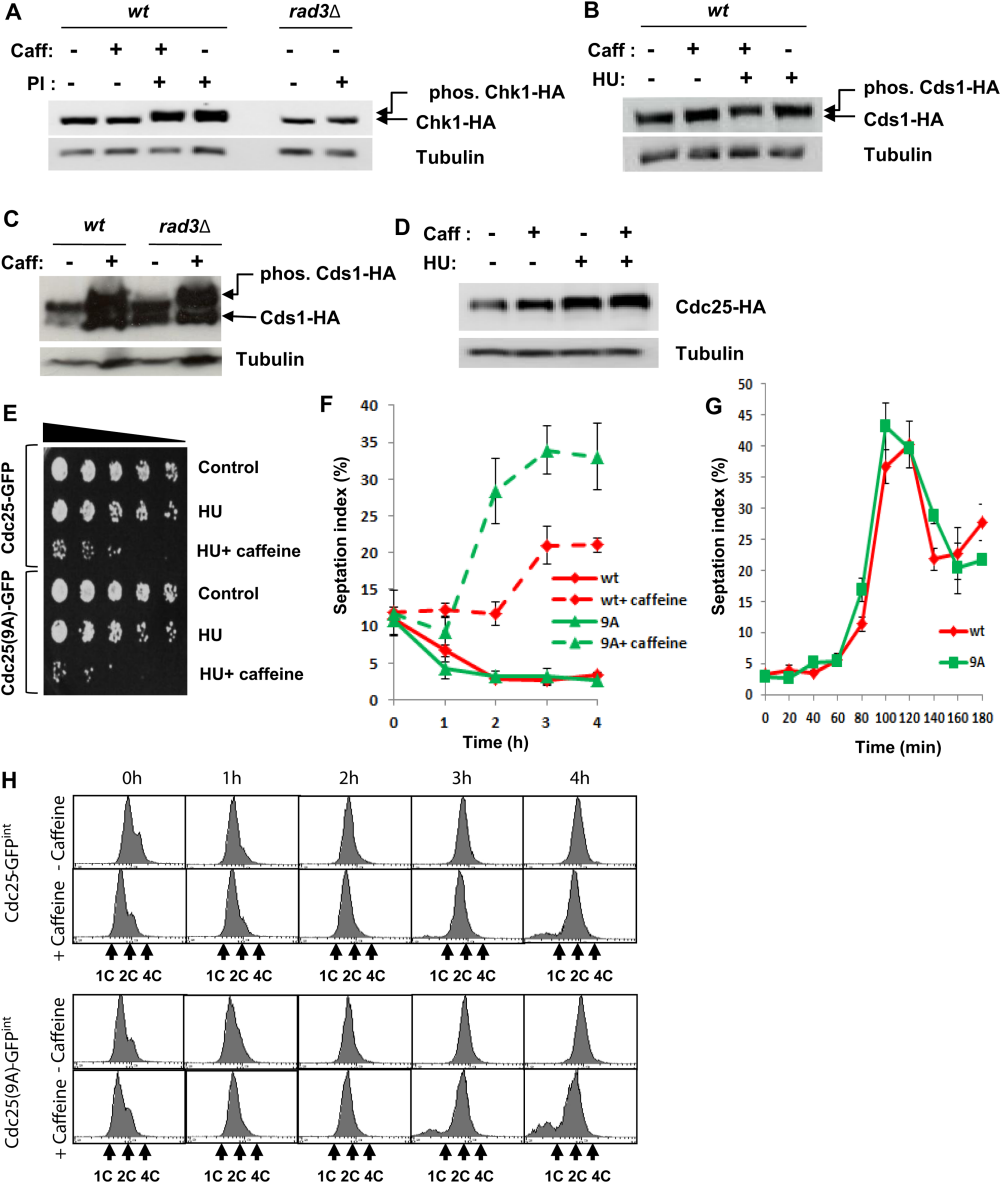
Caffeine modulates checkpoint responses independently of Rad3.A. Cells expressing HA-tagged Chk1 were pre-treated with 10 mM caffeine for 30 min and incubated further for another 2.5 h in the presence of 10 μg ml^−1^ phleomycin. Total protein lysates were probed with monoclonal antibodies directed against HA. Tubulin was used to monitor gel loading. Alternatively, *rad3*Δ mutants expressing HA-tagged Chk1 were incubated for 2.5 h in the presence of 10 μg ml^−1^ phleomycin.B. Cells expressing HA-tagged Cds1 were exposed to 20 mM HU and for a further 2 h with or without 10 mM caffeine. Total protein lysates were treated as in A.C. Wt and *rad3*Δ mutants expressing HA-tagged Cds1 were exposed to 10 mM caffeine for 24 h. Total protein lysates were treated as in A.D. Cells expressing HA-tagged Cdc25 were exposed to 20 mM HU and for a further 2 h with or without 10 mM caffeine. Total protein lysates were treated as in A.E. Cdc25–GFP^int^ and Cdc25^(9A)^–GFP^int^ expressing strains were incubated for 3 h with 20 mM HU and then incubated for a further 3 h in the presence or absence of 10 mM caffeine. Equal cell numbers were spotted onto YES agar plates and incubated at 30°C for 3 days.F. Strains in E were incubated with 20 mM HU for 2 h and then for a further 4 h in the presence or absence of 10 mM caffeine. Samples harvested at the indicated time points were stained with aniline blue and the septation index determined by fluorescence microscopy. Error bars represent the mean of at least three independent experiments ± S.E.G. Cdc25–GFP^int^ and Cdc25^(9A)^–GFP^int^ expressing strains were incubated for 3 h with 20 mM HU, washed with sterile distilled water and resuspended in fresh YES media. Samples harvested at the indicated time points were stained with aniline blue and the septation index determined by fluorescence microscopy. Error bars represent the mean of at least three independent experiments ± S.E.H. Strains in F were analysed by FACS.

Our findings on the differential impact of caffeine on cell cycle progression in *cdc2-3w* and *cdc2-3w cdc25*Δ mutants suggested a central role for Cdc25 in mediating these effects. The theory that caffeine overrides cell cycle checkpoints by inhibiting the ATM/Rad3-mediated phosphorylation of downstream targets remains controversial (Cortez, 2003). Furthermore, a direct inhibition of the Rad3-mediated phosphorylation of Cds1 or Chk1 in *S. pombe* has not been demonstrated (Wang *et al*., 1999; Moser *et al*., 2000). We thus re-examined the effect of caffeine on Rad3-mediated signalling following exposure to HU or phleomycin. Interestingly, 10 mM caffeine did not abolish Chk1 phosphorylation in *S. pombe* cells exposed to 10 μg ml^−1^ phleomycin, whereas *rad3*Δ deletion completely abolished phleomycin-induced Chk1 phosphorylation, as expected (Fig. 6A). Similarly, 10 mM caffeine did not inhibit Cds1 phosphorylation following exposure to 20 mM HU (Fig. 6B). Phosphatase treatment of immunoprecipitated HA-tagged Cds1 demonstrated that the kinase is constitutively phosphorylated in log-phase cells (Fig. 6C and Supplementary Fig. S6A). This phosphorylation was not abolished in *rad3*Δ mutants although an increase in the levels of hypo-phosphorylated Cds1 was clearly detectable (Fig. 6C). Prolonged exposure (24 h) to 10 mM caffeine resulted in a pronounced increase of both Cds1 isoforms in both wt and *rad3*Δ mutants (Fig. 6C). Hence, caffeine does not inhibit Cds1 phosphorylation and induces its accumulation independently of Rad3. Similar results were observed with Chk1 in wt and *rad3*Δ cells (Supplementary Fig. S6B).

Caffeine stabilizes Cdc25 in *S. pombe* (Fig. 1). Since caffeine appeared not to inhibit Rad3 signalling (Fig. 6A–C), we hypothesized that caffeine might induce Cdc25 levels above the threshold required to maintain checkpoint activation. The levels of Cdc25 in cells co-exposed to 20 mM HU and 10 mM caffeine were higher than in cultures exposed to either agent alone (Fig. 6D and Supplementary Fig. S6C). We next compared the effect of caffeine on replication checkpoint activation in strains expressing Cdc25–GFP^int^ and Cdc25^(9A)^–GFP^int^ (Frazer and Young, 2011; 2012) (Supplementary Fig. S6D) expressing strains. Both strains were incubated with 20 mM HU for 2 h, exposed to 10 mM caffeine and the kinetics of cell cycle progression investigated. The ability of caffeine to override the replication checkpoint in Cdc25^(9A)^–GFP^int^ cultures was modestly enhanced relative to Cdc25–GFP^int^ as measured by cell cycle progression (Fig. 6H), but barely as measured by cell survival (Fig. 6E). The rate of mitotic progression and septation index were also higher in Cdc25^(9A)^–GFP^int^ cells exposed to HU and caffeine (Fig. 6F). These results were similar to those observed in experiments using *srk1*Δ mutants (Fig. 5A–C). The sensitivity to HU and degree of chromosome missegregation in Cdc25^(9A)^–GFP^int^ mutants in the presence of caffeine was also greater than observed in Cdc25–GFP^int^ cells (Fig. 6E and H). In contrast, when Cdc25–GFP^int^ and Cdc25^(9A)^–GFP^int^ cells were grown in 20 mM HU for 4 h and then transferred into fresh rich media, no difference in the kinetics of cell cycle re-entry was observed (Fig. 6G).

**Figure 7:**
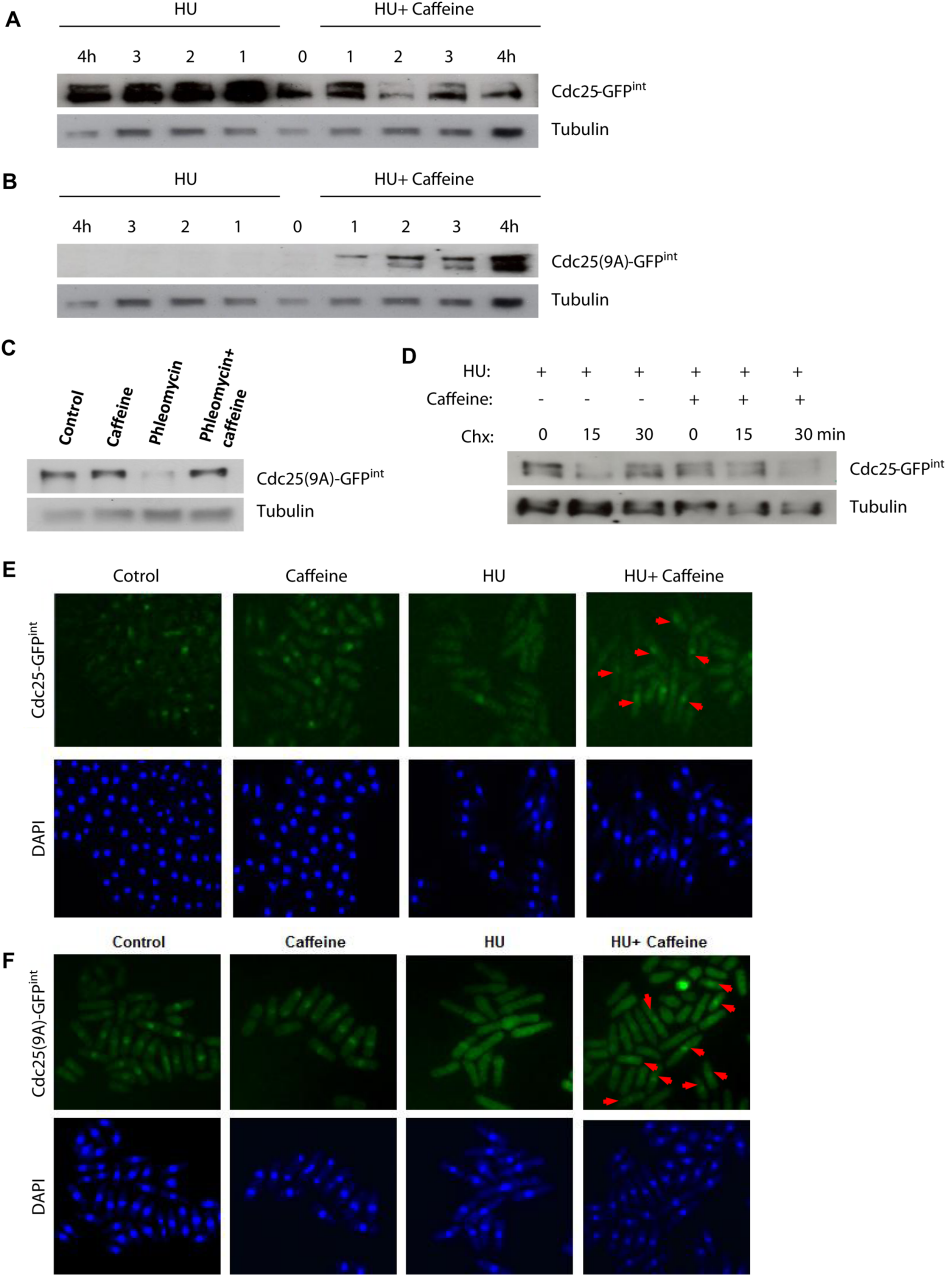
Caffeine stabilizes Cdc25 in the presence of HU.A and B. Cdc25–GFP^int^ and Cdc25^(9A)^–GFP^int^ expressing strains were incubated with 20 mM HU with or without 10 mM caffeine and harvested at the indicated time points. Total protein lysates were probed with monoclonal antibodies directed against GFP. Tubulin was used to monitor gel loading.C. Cdc25^(9A)^–GFP^int^-expressing cells were pre-treated with 10 mM caffeine and then incubated for a further 1.5 h in the presence of 10 μg ml^−1^ phleomycin. Total protein lysates were treated as in A.D. Cdc25–GFP^int^-expressing cells were cultured for 2 h in the presence of 20 mM HU and then for 1 h in the presence or absence of 10 mM caffeine. Cells were then exposed to 100 μg ml^−1^ of Chx and harvested at the indicated time points. Total protein lysates were probed with monoclonal antibodies directed against GFP. Tubulin was used to monitor gel loading.E and F. Cdc25–GFP^int^ and Cdc25^(9A)^–GFP^int^ expressing strains were incubated for 3 h with 20 mM HU and then incubated for a further 1 h in the presence or absence of 10 mM caffeine. Cells were fixed, stained with antibodies against tubulin and examined by fluorescence microscopy. Nuclei were stained using DAPI. Red arrows indicate cells with predominantly nuclear Cdc25 localization.

On exposure to 20 mM HU, Cdc25–GFP^int^ becomes phosphorylated, is exported from the nucleus and accumulates (stockpiles) in the cytoplasm. In contrast, Cdc25^(9A)^–GFP^int^ cannot be phosphorylated and is degraded within the nucleus (Frazer and Young, 2011; 2012) (Fig. 7A and B). Co-exposure to 10 mM caffeine suppressed the accumulation of Cdc25–GFP^int^ (Fig. 7A). In contrast, co-exposure to caffeine clearly induced the accumulation of Cdc25^(9A)^–GFP^int^. Caffeine similarly suppressed the degradation of Cdc25^(9A)^–GFP^int^ in cells exposed to 10 μg ml^−1^ phleomycin (Fig. 7C). Microscopic analyses of Cdc25–GFP^int^ and Cdc25^(9A)^–GFP^int^ expressing strains, demonstrated increased nuclear levels of Cdc25–GFP in cells co-exposed to HU and caffeine relative to cells exposed to HU alone (Fig. 7E and F). The ability of caffeine to override checkpoint signalling was cAMP independent, as *pka1*Δ mutants, which lack the catalytic subunit of protein kinase A, are sensitized to HU by caffeine (Supplementary Fig. S6E). Similarly, Mik1 was not required for the effect of caffeine on DNA damage checkpoints (Supplementary Fig. S6E). Caffeine thus appears to stabilize Cdc25 by suppressing the rate of its degradation within the nucleus. Indeed, the Cdc25–GFP in cells exposed to HU alone appeared to be more stable than Cdc25–GFP co-exposed to HU and caffeine (Fig. 7A). These observations suggest that caffeine interferes with both the nuclear export and degradation of Cdc25. This may explain the failure of caffeine to induce Cdc25–GFP^int^ accumulation in the presence of HU (Fig. 7A). Together these findings provide further evidence that caffeine overrides cell cycle checkpoints by inducing the nuclear accumulation of Cdc25.

## Discussion

In the current study, we have investigated the effect of caffeine on Cdc25 stability and its impact on the cell cycle kinetics of *S. pombe*. Caffeine has generated much interest due to its ability to override checkpoint signalling in both yeast and mammalian cells (Bode and Dong, 2007). Previous studies in *S. pombe* and mammalian cells have proposed that caffeine overrides checkpoint signalling by inhibiting Rad3 and its mammalian homologues ATM and ATR (Wang *et al*., 1999; Moser *et al*., 2000; Zhou *et al*., 2000). The assertion that caffeine overrides checkpoint signalling by inhibiting Rad3 or ATM and ATR remains controversial. Caffeine has been shown to override checkpoint signalling without inhibiting ATM or ATR in mammalian cells (Cortez, 2003). Caffeine has previously been reported to stabilize Cdc25A in mammalian cells. This study also demonstrated the constitutive regulation of Cdc25A by ATR and Chk1 (Sørensen *et al*., 2004). Herein, we have demonstrated for the first time that caffeine stabilizes Cdc25 in *S. pombe* independently of Rad3 and Cds1 (a functional homologue of mammalian Chk1). A recent study reported that caffeine induces Sty1 activation in *S. pombe* (Calvo *et al*., 2009). Sty1 regulates Cdc25 stability and activity (Shiozaki and Russell, 1995; Kishimoto and Yamashita, 2000; Lopez-Aviles *et al*., 2005) but the impact of this activity on caffeine-induced checkpoint override has not been previously reported. Herein we have demonstrated that Sty1 and Mad2 attenuate the ability of caffeine to override checkpoint signalling. The current model of caffeine–induced checkpoint override thus needs to be modified to include its effects on Cdc25 stability and activity.

### Effect of caffeine on Cdc25 stability

Exposure to caffeine resulted in rapid accumulation of Cdc25 in *S. pombe*. In mammalian cells, caffeine or the inhibition of ATR-Chk1 signalling has similarly been shown to induce the stabilization of Cdc25A. Caffeine has similarly been shown to stabilize Cdc25B in mammalian cells (Varmeh and Manfredi, 2009). Furthermore, Chk1 has been shown to regulate Cdc25A activity and mitotic entry even during the normal cell cycle (Shiromizu *et al*., 2006; Enomoto *et al*., 2009; Matsuyama *et al*., 2011). Our findings demonstrate that *rad3* or *cds1* deletion similarly stabilizes Cdc25 in *S. pombe*. Interestingly, caffeine stabilized Cdc25 in both *rad3* and *cds1* mutants, demonstrating that this stabilization is not due to the inhibition of Rad3 signalling. The levels of *cdc25^+^* mRNA were suppressed in caffeine treated wt cells as well as in *rad3* and *cds1* mutants. Exposure to caffeine or the deletion of *rad3^+^* (or *cds1^+^*) thus stabilizes Cdc25 at the post-translational level, albeit by different mechanisms. In *S. pombe*, Cdc25 degradation is mediated by Pub1 and the anaphase-promoting complex/cyclosome (APC/C). Furthermore, the Clp1-mediated dephosphorylation of Cdc25 is required for its rapid degradation as cells exit mitosis (Wolfe and Gould, 2004; Esteban *et al*., 2008). Caffeine may thus interfere with any of these pathways. Recent studies have demonstrated that the accumulation of Cdc25 does not affect cell size in *S. pombe* (Frazer and Young, 2011; 2012). In our studies, the accumulation of Cdc25 in response to caffeine exposure or deletion of *rad3^+^* (or *cds1^+^*) was likewise not associated with reduced cell size. Mutant isoforms of Cdc25 (Cdc25^(9A)^–GFP^int^) that cannot be phosphorylated are unstable and degraded in a Mik1-dependent manner following activation of the replication or G2 checkpoints (Frazer and Young, 2011; 2012). Caffeine induced Cdc25^(9A)^–GFP^int^ accumulation both in untreated cells and in cells exposed to HU or phleomycin. Our findings suggest that caffeine attenuates the nuclear degradation of Cdc25 in *S. pombe*. Previous findings have shown that Pub1 does not mediate the ubiquitin-dependent degradation of nuclear Cdc25–GFP (Frazer and Young, 2012). The effect of caffeine on Cdc25 stability is thus unlikely to result from the inhibition of Pub1 activity. The APC/C is also believed to mediate the degradation of Cdc25 as cells exit mitosis, and its Clp1-mediated dephosphorylation during exit from mitosis is required for its rapid degradation. The levels of Cdc25 remain elevated in actively growing *S. pombe* cultures exposed to caffeine. Furthermore, Cdc25 is detectable in stationary-phase cells previously exposed to caffeine but not in untreated cultures. These observations suggest that caffeine interferes with the degradation of Cdc25 during mitosis. Caffeine may thus interfere with APC/C-mediated Cdc25 degradation. Alternatively, caffeine may stabilize Cdc25 by inhibiting its dephosphorylation. Future studies will address the roles of Rad3 and Cds1 in regulating Cdc25 stability in normally cycling cells.

### Effect of caffeine on cell cycle kinetics

We have noted with interest that the precise effect of caffeine on the cell cycle kinetics of *S. pombe* is strongly influenced by mutations that positively affect Cdc2 activity. On entry into mitosis, the suppression of Cdc25 and Cdc2 activity is required for mitotic exit and progression through cytokinesis (Wolfe and Gould, 2004; Esteban *et al*., 2008). Exposure to caffeine induced Cdc25 accumulation and a significant reduction in the level of Cdc2 Tyr15 phosphorylation even in normally cycling cells (Fig. 1I). The minimal effect of caffeine on the cell cycle kinetics of wt cells was likely due to the cells’ ability to counteract the increase in Cdc25 activity. Our findings demonstrate for instance that Rad3 and Cds1 negatively regulate Cdc25 stability during the normal cell cycle. Furthermore, the positive effects of caffeine-induced Sty1 activation on Cdc25 activity and cell cycle progression are countered by the simultaneous activation of Srk1 (reviewed in Alao and Sunnerhagen, 2008). Accordingly, exposure to caffeine substantially influenced the rate of cell cycle progression in *cdc2-3w*, *cds1*Δ, *rad3*Δ, *srk1*Δ and *wee1*Δ mutants (Figs 2, 4 and 5). These mutants are unable to effectively negatively regulate Cdc2 activity. Exposure of *wee1*Δ mutants to caffeine clearly promoted progression through S phase, indicating that caffeine can positively influence cell cycle progression. Exposure of *cdc2-3w*, *cds1*Δ and *rad3*Δ mutants to caffeine was also associated with a rapid increase in the population of septating cells. Recent studies have demonstrated that the activity and localization, rather than its expression level, determine the ability of Cdc25 to promote entry into mitosis (Frazer and Young, 2011; 2012). The precise impact of caffeine-induced Cdc25 accumulation on cell cycle progression is thus influenced by the genetic background of the exposed strain. Caffeine-induced Cdc25 accumulation likely advances entry into mitosis but delays progression through cytokinesis as a consequence (Wolfe and Gould, 2004; Esteban *et al*., 2008). Careful analyses of the effects of caffeine on cell cycle progression in these mutants demonstrated that caffeine indeed delays progression through mitosis and cytokinesis. Crucially, Cdc25 expression was required for cell cycle progression in the presence of caffeine. Activation of the *cdc2-3w* allele occurs independently of Cdc25 but is still subject to negative regulation by Wee1. Cdc25 thus continues to influence cell cycle progression in *cdc2-3w* mutants (Enoch *et al*., 1992; Basi and Enoch, 1996). Exposure of *cdc2-3w* mutants to caffeine was associated with a decreased rate of progression through mitosis. In contrast, cell cycle progression was inhibited when *cdc2-3w cdc25*Δ mutants were exposed to caffeine. We also observed that exposure to caffeine suppressed Tyr15 phosphorylation on Cdc2 in *cdc2-3w* but not *cdc2-3w cdc25*Δ mutants. Our study demonstrates that caffeine positively modulates cell cycle progression by inducing Cdc25 accumulation. Consequently, caffeine delays cell cycle progression and enhances resistance to HU in *cdc2-3w cdc25*Δ mutants. This effect on cell cycle progression is however strongly attenuated in wt cells exposed to caffeine under normal conditions.

### Caffeine modulates spindle checkpoint activation

Exposure to caffeine suppressed the requirement for the spindle checkpoint in wt and *mad2*Δ mutants following microtubule depolymerization. Cell cycle analyses demonstrated that caffeine delays progression through cytokinesis thus delaying the chromosome missegregation that would otherwise occur. Following exposure to MBC at 30°C, the spindle checkpoint is only partially able to prevent progression through mitosis (Castagnetti *et al*., 2010). Interestingly, caffeine was more effective at suppressing MBC-induced chromosome missegregation in wt cells than in *mad2*Δ mutants. Furthermore, *mad2*Δ mutants were clearly advanced through mitosis and S phase relative to wt cells following exposure to caffeine alone. Caffeine was also more effective at suppressing resistance to HU in *mad2*Δ mutants than in wt cells. Our studies clearly demonstrate that caffeine exerts both positive and negative effects on cell cycle progression in *S. pombe*. They also suggest that Mad2 and the spindle checkpoint suppress the ability of caffeine to promote cell cycle progression. We and others have previously demonstrated that activation of the stress response pathway interferes with spindle dynamics and partially delays cell cycle progression in a Mad2-dependent manner (Tatebe *et al*., 2005; Kawasaki *et al*., 2006; Robertson and Hagan, 2008; Alao *et al*., 2010). It is thus likely that caffeine interferes with satisfaction of the spindle checkpoint, resulting in sustained Mad2 activation and delayed progression through mitosis. Sustained inhibition of the APC/C following exposure to caffeine may also account in part for the accumulation of Cdc25. Paradoxically, caffeine can also compensate for the loss of the spindle checkpoint in *mad2*Δ mutants by delaying progression through cytokinesis.

### Sty1 modulates caffeine activity

Sty1 is a key regulator of the ESR and has been shown to enhance Cdc25 activity (Shiozaki and Russell, 1995; Kishimoto and Yamashita, 2000). However, Sty1 can also negatively regulate Cdc25 activity *via* activation of Srk1. It has been previously demonstrated that exposure to osmotic stress induces Cdc25 accumulation and delays cell cycle progression in part through activation of Srk1 (Tatebe *et al*., 2005; Kawasaki *et al*., 2006; Robertson and Hagan, 2008; Alao *et al*., 2010). Following exposure to osmotic stress, Srk1 phosphorylates Cdc25 targeting it for nuclear export (Lopez-Aviles *et al*., 2005). The accumulation or ‘stockpiling’ of Cdc25 has been observed under various conditions and is dependent on Sty1-induced Srk1 activation (Kovelman and Russell, 1996; Kishimoto and Yamashita, 2000; Lopez-Aviles *et al*., 2005; Alao *et al*., 2010; Frazer and Young, 2011; 2012). The stockpiling of Cdc25 may facilitate rapid resumption of cell cycle progression following adaptation to stress or DNA damage repair (Kovelman and Russell, 1996; Degols and Russell, 1997). Srk1 thus facilitates the stockpiling of Cdc25 while simultaneously inhibiting its ability to promote cell cycle progression. Srk1 also negatively regulates Cdc25 activity during the normal cell cycle (Lopez-Aviles *et al*., 2005). Exposure to caffeine induces activation of Sty1 (Calvo *et al*., 2009). We predicted that the simultaneous induction of Cdc25 accumulation and activation of Sty1–Srk1 signalling by caffeine would inhibit its ability to positively mediate entry into mitosis. In our studies, deletion of *srk1^+^* only modestly influenced the effect of caffeine on cell cycle progression relative to wt cells (Supplementary Fig. S2A and B). In contrast, the ability of caffeine to override the replication checkpoint was greatly enhanced in *srk1*Δ mutants. Consequently, *srk1*Δ mutants showed increased chromosome missegregation and sensitivity when exposed to HU and subsequently caffeine. Srk1 thus attenuates the ability of caffeine to override checkpoints, presumably by inhibiting Cdc25 activity. We have previously shown that exposure to osmotic stress delays cell cycle progression in an Srk1-dependent manner (Alao *et al*., 2010). Exposure to potassium chloride or caffeine induces Cdc25 accumulation in *S. pombe*. Srk1 thus appears to play a crucial role in counteracting Cdc25 activity under these conditions.

### Effect of caffeine on checkpoint signalling

Previous studies have proposed that caffeine overrides checkpoint signalling in *S. pombe* by inhibiting Rad3 (Wang *et al*., 1999; Moser *et al*., 2000). These studies have provided biochemical evidence that caffeine does indeed override checkpoint signalling in this organism. Caffeine inhibits HU-induced Chk1 phosphorylation in *cds1*Δ mutants (Moser *et al*., 2000). The direct inhibition of Cds1 or Chk1 phosphorylation in S phase and G2 respectively has however not been demonstrated. Interestingly, caffeine did not inhibit Rad3-induced Cds1 or Chk1 phosphorylation in our studies. Caffeine has similarly been shown to override checkpoint signalling independently of ATR inhibition in mammalian cells (Cortez, 2003). We have demonstrated here that caffeine induces Cdc25 accumulation in *S. pombe*. The inhibition of Cdc25 activity is essential for full activation of the replication and DNA damage checkpoints (Furnari *et al*., 1997; 1999; Boddy *et al*., 1998; Zeng *et al*., 1998). Intriguingly, caffeine did not inhibit Rad3 induced Cds1 or Chk1 phosphorylation following exposure to HU or phleomycin. Since caffeine suppresses Cdc2 phosphorylation under these conditions, its positive effect on Cdc25 stability may underlie its ability to override checkpoints. Cdc25A degradation is required for S phase arrest in mammalian cells (Mailand *et al*., 2000; Jin *et al*., 2003). Our findings clearly demonstrate that caffeine induces the nuclear accumulation of Cdc25. Cdc2 and Cdc13 are highly enriched in the nucleus of S phase arrested and late G2 phase cells (Decottignies *et al*., 2001). Caffeine may thus lead to inappropriate Cdc2 activation during cell cycle arrest. The deletion of *srk1^+^* or expression of Cdc25^(9A)^–GFP^int^ had little effect on the rate of cell cycle progression in cells exposed to caffeine. In contrast, the ability of caffeine to override checkpoint signalling was greatly enhanced in these mutants. Furthermore, Cdc25–GFP^int^ and Cdc25^(9A)^–GFP^int^ re-entered the cell cycle with similar kinetics when released from a HU block. We have also observed that Cdc25 expression is necessary for the positive effects of caffeine on cell cycle progression. These findings provide further evidence that Cdc25 accumulation and not Rad3 inhibition underlies the caffeine-induced checkpoint override.

### Conclusions

We have demonstrated for the first time that caffeine induces Cdc25 stabilization independently of Rad3 in *S. pombe*. Similarly, *rad3* or *cds1* deletion stabilized Cdc25, suggesting a constitutive role in regulating its stability. Cdc25 expression is required for mitotic progression in *S. pombe* cells exposed to caffeine. Srk1 and Mad2 attenuate the ability of caffeine to drive cells through mitosis. Furthermore, caffeine-induced Cdc25 accumulation delays progression through cytokinesis. Caffeine did not inhibit Rad3 signalling in cells exposed to HU or phleomycin. Our findings suggest strongly that caffeine at least partially overrides checkpoint signalling by stabilizing Cdc25.

## Experimental procedures

### Strains, media and reagents

Strains are listed in Table 1. Cells were grown in yeast extract plus supplements medium (YES). Stock solutions of caffeine (Sigma Aldrich AB, Stockholm, Sweden) (100 mM) were prepared in water stored at −20°C. HU (Sigma Aldrich AB) was dissolved in water at a concentration of 1 M and stored at −20°C. Phleomycin (Sigma Aldrich AB) was dissolved in water and stock solutions (10 μg ml^−1^) stored at −20°C. MBC (Carbendazim/methylbenzimidazol-2yl carbamate) and latrunculin B (Lat B) (Sigma Aldrich AB) were stored at −20°C as 10 mg ml^−1^ stock solutions in DMSO.

**Table 1:** *S . pombe* strains

*h^−^ L972*	Lab stock
*cds1::ura4^+^*	H. Okayama
*h^−^ chk1::KanMX6*	This study
*h^−^ leu1 ura4 his3 srk1::ura4^+^*	Lab stock
*h^−^ wee1::ura4^+^ leu1-32 ura4-D18* **(FY7283)**	YGRC
*h^−^ cdc2-3w* **(FY8156)**	YGRC
*h^−^ cdc2-3w cdc25::ura4^+^ leu1-32 ura4-D18*	YGRC
*h^−^ Chk1:ep leu1-32 ade6-216*	N. Walworth
*h^−^ Chk1:ep leu1-32 ade6-216 rad3::KanMx6*	This study
*h^−^ leu1 ura4 cds1-2HA6His[ura4^+^]* **(FY11064)**	YGRC
*h^−^ leu1 ura4 cds1-2HA6His[ura4^+^] rad3::KanMx6*	This study
*h^+^ cdc25-6HA [ura4^+^] leu1-32 ura4-D18* **(FY7031)**	YGRC
*h^+^ cdc25-6HA [ura4^+^] leu1-32 ura4-D18 rad3::KanMx6*	This study
*h^+^ cdc25-6HA [ura4^+^] leu1-32 ura4-D18 rad24::KanMx6*	This study
*h^+^ cdc25-6HA [ura4^+^] leu1-32 ura4-D18 cds1::KanMx6*	This study
*h^−^ ura4-D18 leu1-32 cdc25-12myc::ura4^+^*	P. Russell
*h^+^ leu1 ura4-D18 pka1::ura4^+^ his2* **(FY10302)**	YGRC
*h^−^ leu1 cut2-364* **(FY11545)**	YGRC
*h^+^ leu1 nda3-KM311*	A. Bueno
*h^−^ nda3-KM311 mad2::ura4 leu1-32 ura4-D18*	S. Sazer
*h^+^ leu1 nda3-KM311 cdc25-myc::ura4^+^ ura4D18 ade6M21X leu1-32*	K. Gould
*h^+^ leu1 nda3-KM311 cdc25-myc::ura4^+^ clp1Δ ura4-D18 ade6-M21X leu1-32*	K. Gould
*h^−^ cdc25–GFP^int^ cdc25::ura4^+^ ura4-D18 leu1-32*	P. Young
*h^+^ cdc25^(9A)^–GFP^int^ cdc25::ura4^+^ ura4-D18 leu1-32*	P. Young
*h^+^ srk1-HA::Kan^+^ ura4-D18 leu1-32*	R. Aligue
*h^−^ mik1::ura4 leu1 ura4* **(FY8317)**	YGRC

YGRC, Yeast Genetic Resource Center, Osaka, Japan.

### Molecular genetics

Deletion of open reading frames was done by PCR-based genomic targeting using a *KanMX6* construct (Bähler *et al*., 1998). Disruptions were verified by screening for UV or HU sensitivity (where appropriate) followed by PCR using genomic DNA extracted from mutants with the expected UV- or HU-sensitive phenotype.

### Microscopy

Aniline blue staining and septation index assays were carried out as previously described (Kippert and Lloyd, 1995; Dunaway and Walworth, 2004; Forsburg and Rhind, 2006). Images were obtained with a Zeiss AxioCam on a Zeiss Axioplan 2 microscope with a 100× objective using a 4,6-diamidino-2-phenylindole (DAPI) filter set. Strains expressing recombinant GFP constructs were fixed in methanol at −20°C. Fixed cells were mounted in VECTASHIELD® mounting medium and visualized using differential interference contrast (DIC) or a GFP filter set.

### Fluorescence-activated cell sorting (FACS)

Approximately 10^7^ cells were harvested at the desired time points, resuspended in 70% ethanol and stored at 4°C until use. FACS analyses were performed according to the protocol of Sazer and Sherwood (1990), using propidium iodide (32 μg ml^−1^) as outlined on the Forsburg lab page (http://www-bcf.usc.edu/~forsburg/yeast-flow-cytometry.html). Flow cytometry was performed with a BD FACSAria™ cell sorting system (Becton Dickinson AB, Stockholm, Sweden).

### Immunoblotting

Monoclonal antibodies directed against GFP, HA, and Myc were from Santa Cruz Biotechnology (Heidelberg, Germany). Polyclonal antibodies directed against phospho-(Ser317) Chk1 and mouse monoclonal antibodies directed against phospho-(Thr180/Tyr182) p38 were from Cell Signaling Technology [Bionordika (Sweden) AB, Stockholm, Sweden]. Monoclonal antibodies directed against α-tubulin and phospho-(Tyr15) Cdc2 (Cdk1) were from Sigma-Aldrich (Sigma Aldrich AB). Monoclonal antibodies against Cdc2 were from Abcam (Cambridge, UK). For immunoblotting, protein extracts were prepared as previously described (Asp and Sunnerhagen, 2003) with the addition of 1× PhosStop phosphatase inhibitor cocktail (Roche Diagnostics Scandinavia AB, Bromma, Sweden). Proteins were separated by SDS-PAGE. Epitope-tagged proteins were detected with the appropriate monoclonal antibodies.

### Immunoprecipitation and phosphatase assays

For immunoprecipitation of HA-tagged Cds1, protein extractions were performed as previously described (Asp and Sunnerhagen, 2003). Lysates were incubated with 2 μg of anti-HA (F-7) antibody overnight at 4°C, followed by further incubation with 50 μl of Protein A/G agarose (Santa Cruz Biotechnology) for 1 h. Immunoprecipitated HA-tagged Cds1 was treated with λ protein phosphatase as previously described with the exception that 1× PhosStop phosphatase inhibitor cocktail was added as appropriate (Asp and Sunnerhagen, 2003).

### RT-PCR

Early- to mid-log-phase cells were harvested by centrifugation, washed once in water and snap-frozen. Total RNA was extracted using the RiboPure™-Yeast kit (Ambion) according to the instructions. DNase I-treated total RNA was subsequently used in semi-quantitative and quantitative RT-PCR reactions. A One-Step RT-PCR Kit (Qiagen AB, Sollentuna, Sweden) was used for semi-quantitative RT-PCR reactions according to the manufacturer’s instructions. cDNA was synthesized using a SuperScript ™ III kit (Invitrogen AB, Lidingö, Sweden) according to the manufacturer’s instructions. Quantitative Real-Time PCR was performed by the Core Genomics Facility (University of Gothenburg), using the Power SYB® Green PCR Master Mix. One hundred nanograms of each cDNA and 0.5 mM of the primers were used in reaction volumes of 10 μl. The same primer pairs and annealing temperatures as in the One-Step PCR was used, and PCR for each cDNA sample was performed in triplicate. The specificity of the PCR was checked by comparing the T_m_ of the PCR-product to a sample without template.

The Comparative CT Method was used to analyse the results. The mean threshold cycle obtained for each sample was used to calculate initial *cdc25^+^* mRNA levels normalized against *act1^+^* mRNA, assuming the efficiencies of the PCR reactions were equal. Thereafter the *cdc25^+^* mRNA levels in the *cds1* and *rad3* deletion mutants were compared to wt by the ratio (*cdc25^+^* mRNA level) _sample_/(*cdc25^+^* mRNA level) _control_, where the untreated wt sample served as the control.

## Competing interests

The authors declare that they have no competing interests.

## Authors’ contributions

J.P.A. and P.S. conceived and designed the study. J.P.A. performed experiments, analysed data and wrote the manuscript. J.J.S. performed experiments on Cdc25 expression and stability. J.B. performed experiments on Cdc25 stability, cell cycle kinetics and spindle checkpoint regulation. N.Ö.-Y. performed experiments on Cdc25 stability and the effect of caffeine on the DDR. B.K. performed studies on the regulation of the spindle checkpoint.
