# Supplementary material for: Caffeine stabilizes Cdc25 independently of Rad3 in S chizosaccharomyces pombe contributing to checkpoint override
Source: Mol Microbiol. 2014 Apr 14;92(4):777–96. doi: 10.1111/mmi.12592 (PMC4235345; doi:10.1111/mmi.12592)

Supplementary Figure 1

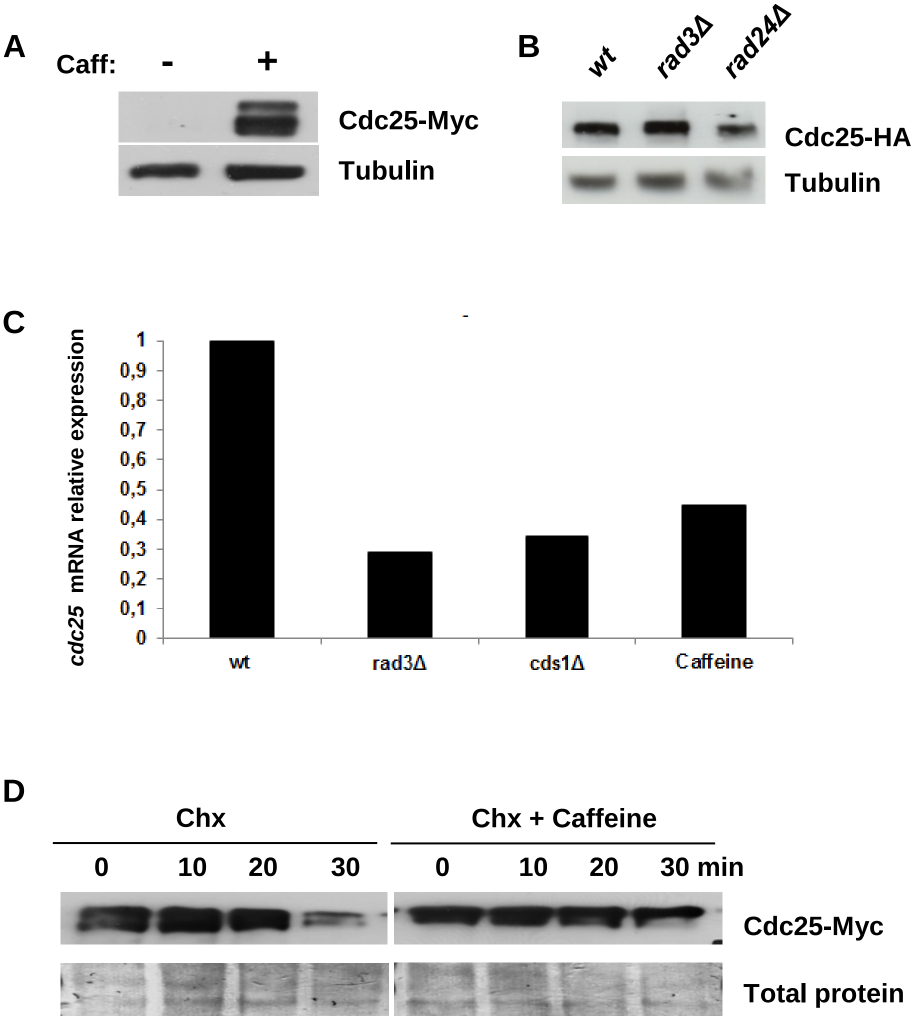

Supplementary Figure 2

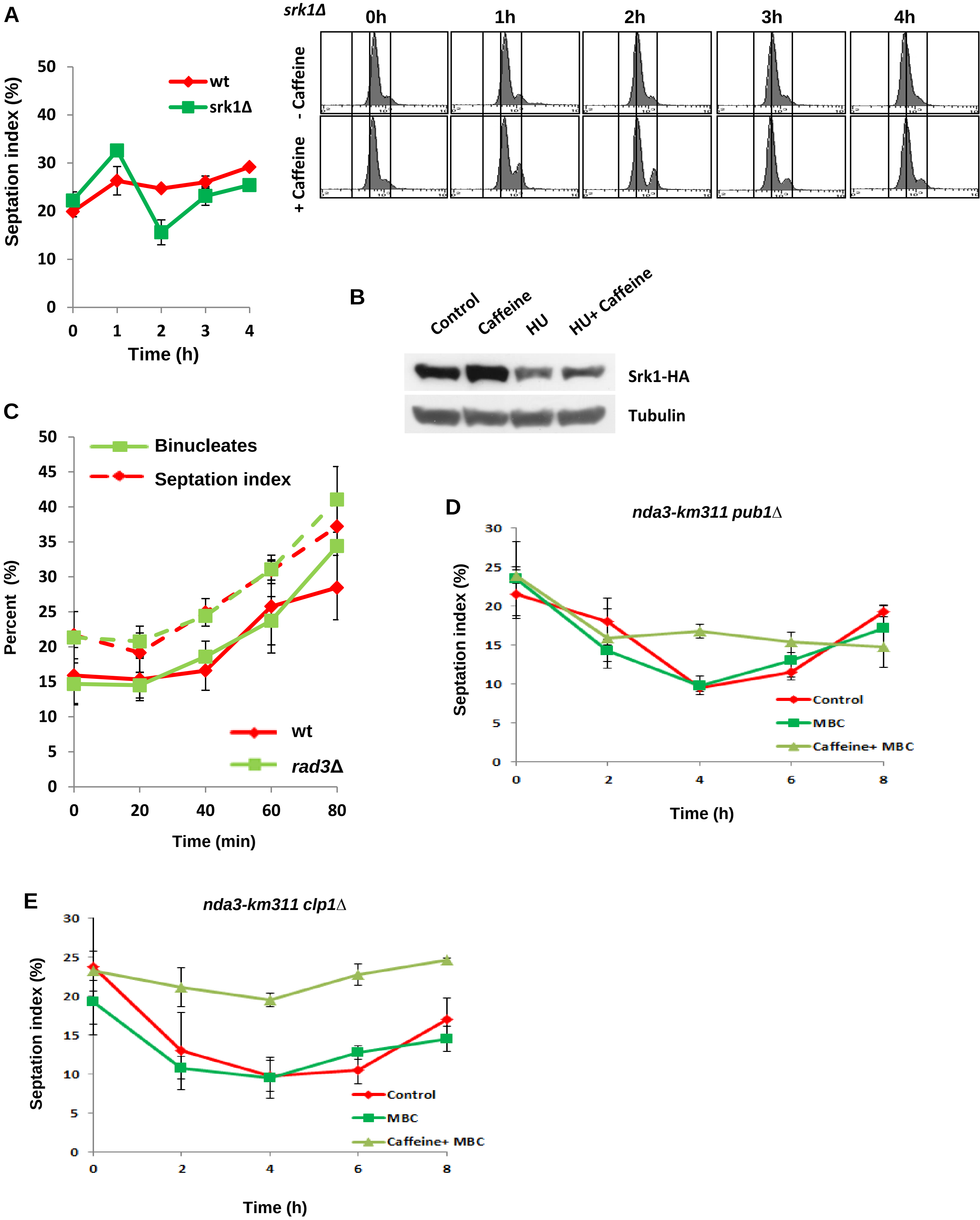

Supplementary Figure 3

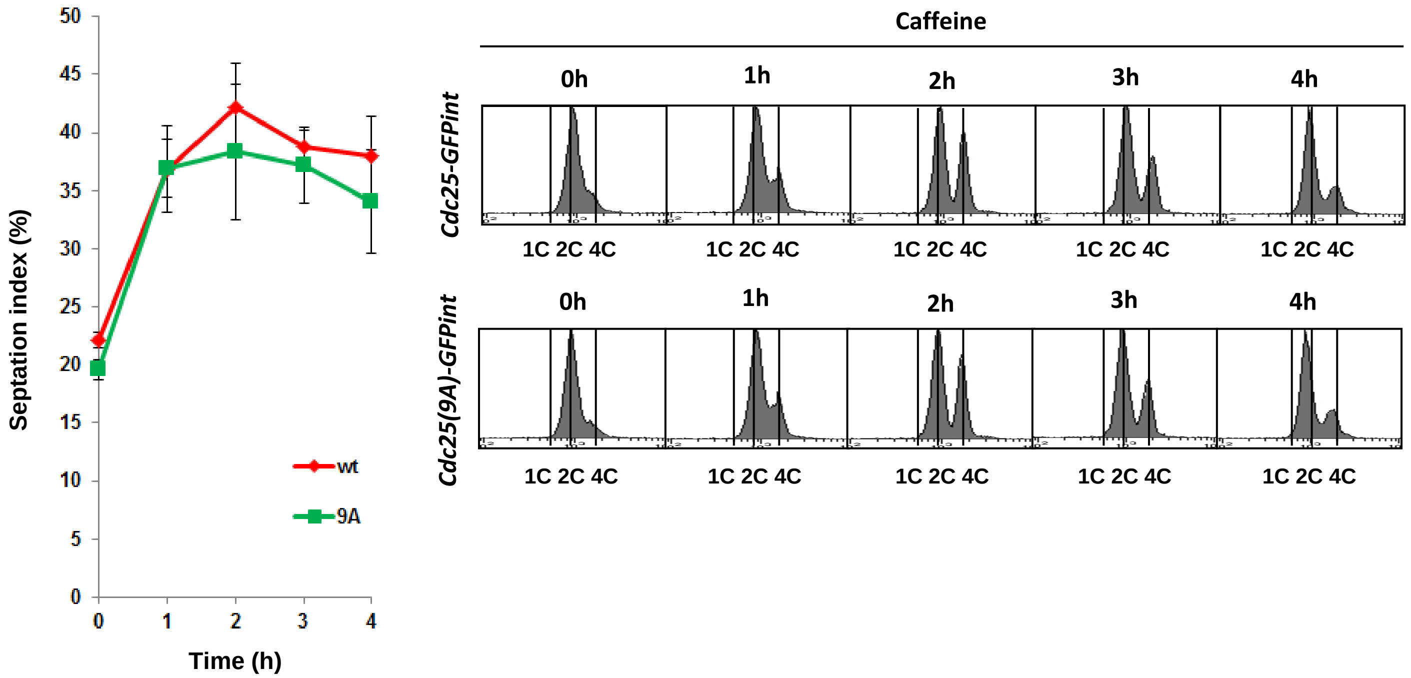

Supplementary Figure 4

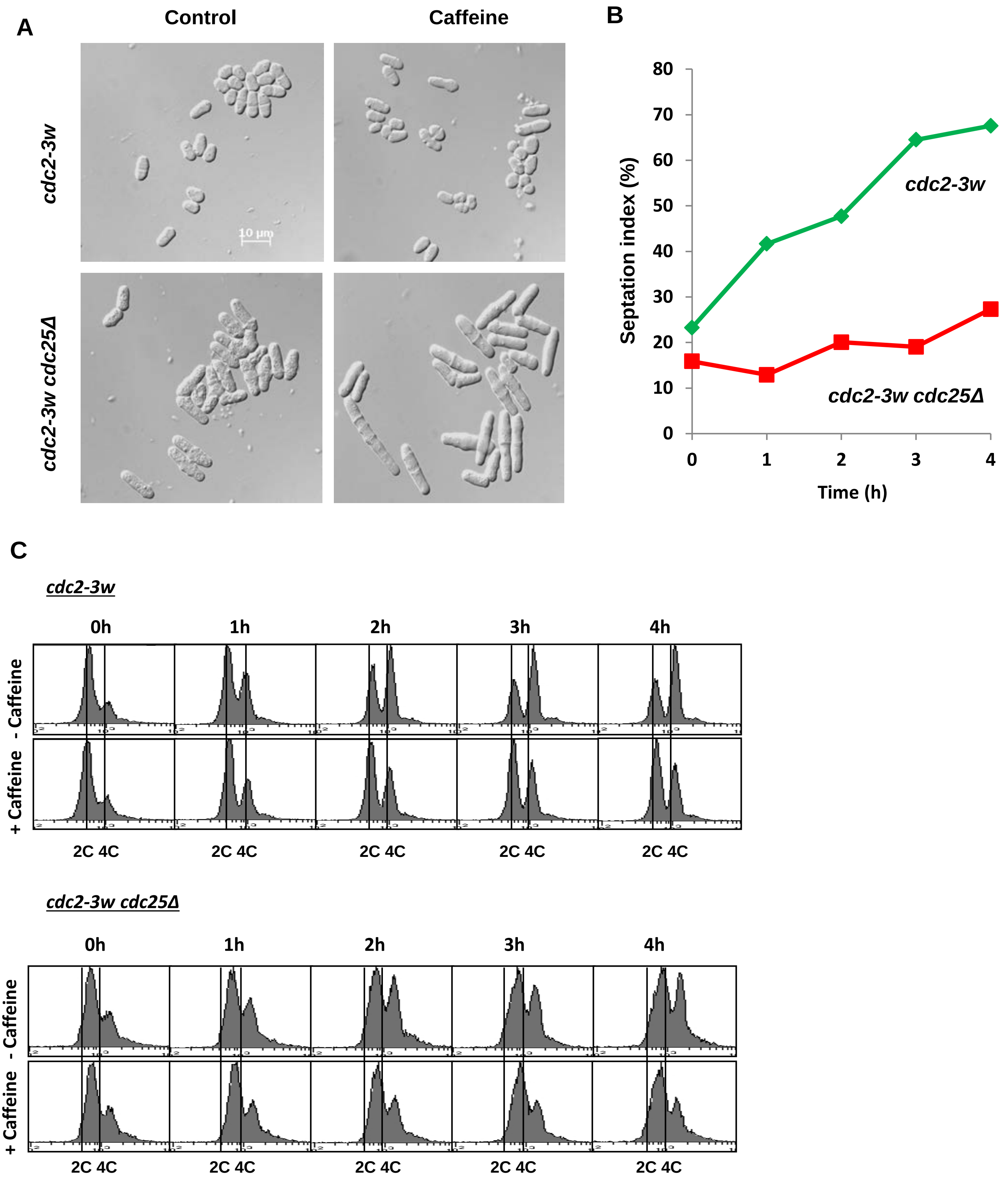

Supplementary Figure 5

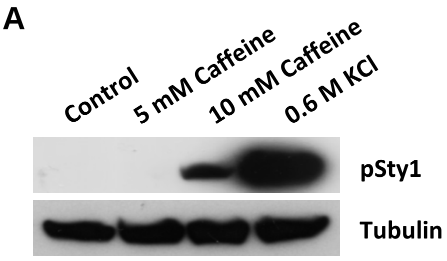

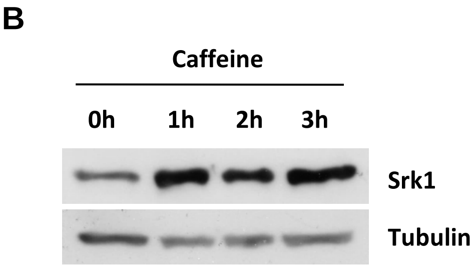

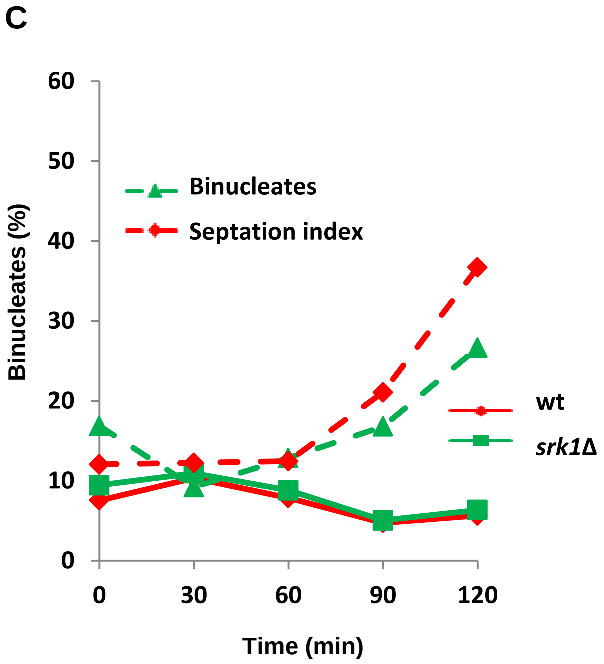

Supplementary Figure 6

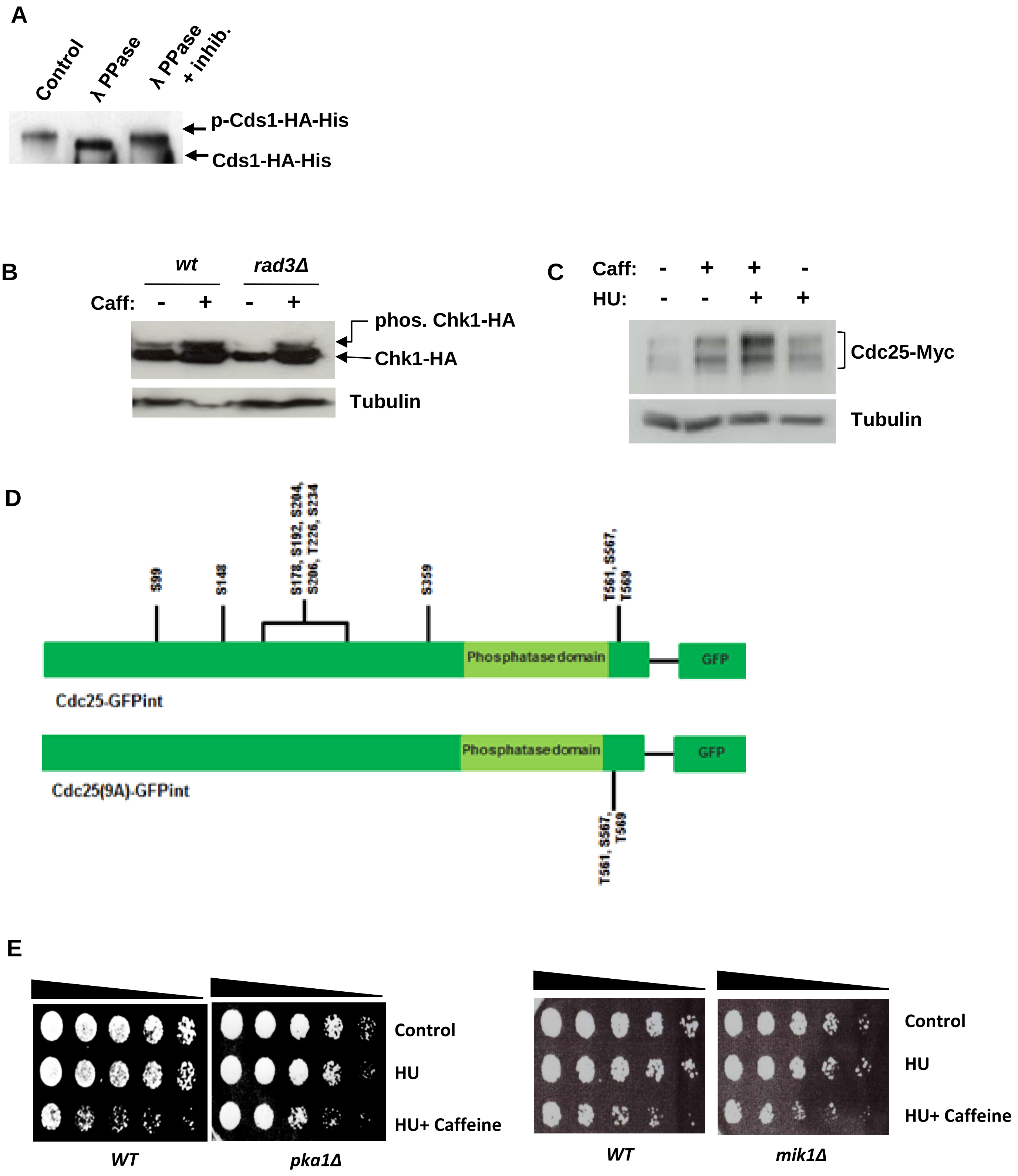

Supplement: Supplementary file 1 [file mmi0092-0777-SD1.pdf]
